# Psychopharmacological interventions among people who use Assisted Reproductive Technology (ART) — a scoping review

**DOI:** 10.1186/s12958-025-01400-4

**Published:** 2025-05-10

**Authors:** Storman Dawid, Jemioło Paweł, Adamska Dominika, Sawiec Zuzanna

**Affiliations:** 1 Epidemiology and Preventive MedicineDepartment of Hygiene and DieteticsFaculty of Medicine, Jagiellonian University Medical College, Krakow, Poland; 2 AGH University of Krakow, Krakow, Poland; 3 Students’ Scientific Group of Systematic Reviews, Jagiellonian University, Krakow, Poland

**Keywords:** Scoping review, Infertility, Assisted reproductive technology, Psychopharmacotherapy, In-vitro fertilization, Intracytoplasmic sperm injection

## Abstract

**Background:**

One in six people experience infertility, often leading couples to seek Assisted Reproductive Technology (ART), which can be emotionally taxing. Anxiety and depression are common among individuals undergoing ART, highlighting the need for integrated mental health support, especially for women who face heightened risk of psychiatric disorders during their reproductive years. Despite the importance of psychiatric care, there is limited knowledge regarding the use of psychiatric medications among individuals undergoing ART.

**Methods:**

Following a pre-written protocol (osf.io/egxw8/), we systematically searched electronic databases (Ovid MEDLINE, Embase, CENTRAL, Web of Science, PsycInfo, Scopus) up to 15.02.2025 to identify any study focused on psychopharmacotherapy among people undergoing ART. Two independent reviewers screened titles, abstracts, and full texts, performed data extraction, and resolved conflicts through discussion or consultation with a third reviewer. We synthesized data using descriptive analysis and performed analysis within the following subgroups: (1) group of medication according to Neuroscience-based Nomenclature (NbN) classes; (2) indication for using a specific medication (psychiatric / non-psychiatric).

**Results:**

We included 29 studies published in 31 papers. Psychiatric medications were primarily administered for the treatment of depression (n=12/29 studies, 41.38%) and anxiety disorders (n=8/29, 27.59%). Among all groups of medication serotonin reuptake inhibitors were the most commonly studied class of medications (n=15/29 studies, 51.72%), with fluoxetine being the most frequently used medication (n=8/29, 27.59%). Among medications for anxiety, GABA Positive Allosteric Modulators with midazolam occurred the most frequently (n=6/29, 20.69%). Medications for psychosis included single drugs: olanzapine, clozapine, risperidone, quetiapine, aripiprazole, haloperidol, and promethazine. Among medications that could be used in bipolar disorder there were: valproic acid, lithium, and lamotrigine.

The most commonly reported endpoints in the studies were psychiatric symptom severity (n=11/29, 37.93%) and ART efficacy (n=10/29, 34.48%).

**Conclusion:**

Evidence on the use of psychopharmacotherapy in ART primarily concerns women. Available literature indicates that psychopharmacotherapy for individuals undergoing ART frequently involves medications commonly used in general psychiatric practice, with a tendency toward lower dosing and a preference for serotonin reuptake inhibitors. However, this observation should be interpreted cautiously, as current evidence remains limited and further research is warranted to establish treatment patterns more conclusively. The variability in study designs and reporting standards further highlights the need for standardized methodologies and improved adherence to reporting guidelines to enhance the quality and applicability of future research.

**Trial registration:**

Review registration number: osf.io/egxw8/

**Supplementary Information:**

The online version contains supplementary material available at 10.1186/s12958-025-01400-4.

## Introduction

Globally, approximately one in six people experience infertility at some point in their lives [56]. Infertility refers to a condition in which a couple experiences difficulty in achieving pregnancy despite regular, unprotected sexual intercourse for an extended period (typically one year). An increasing number of couples are seeking assistance through assisted reproductive technology (ART) to conceive and fulfill their goal of having a healthy child [14]. The WHO report [56] on the prevalence of infertility by region indicates that the highest rate is observed in the Western Pacific region (23.2%) and the lowest in the Eastern Mediterranean region (10.7%). Sun et al. [48] found that the global age-standardized prevalence rate of female infertility increased by almost 15% from 1990 to 2017, while the male infertility rate increased by more than 8% during the same period. Additionally, Liang et al. [28] estimated that in 2021, approximately 55 million men and 110 million women were living with infertility worldwide and predicted that these numbers would increase very rapidly by 2040.

The emotional burden of these treatments is significant, with approximately 30% of couples discontinuing treatment prematurely due to the stress involved [7]. The pressure from societal and familial expectations further intensifies the psychological toll, which might lead to anxiety, depression, or other psychiatric disorders among both men and women.

Emotional distress has been identified as a key reason for discontinuing ART, even in women without prior psychiatric history. The cumulative effect of societal pressure, coupled with the low success rate of in vitro fertilization (IVF) — around 30% per cycle — means many couples face repeated failures, further contributing to psychological strain [57]. Anxiety and depression are widespread among people experiencing ART procedures, with studies reporting rates as high as around 20–30% and 13–15%, respectively [29]. This suggests a critical need for integrating mental health support within ART programs to address the significant emotional burden faced by infertile couples.

Women, in particular, face a heightened risk of depression, especially during their reproductive years, when infertility adds a profound layer of emotional stress. Studies indicate that 17% of women aged 20–44 experience infertility, and many of them seek treatment, which can exacerbate their risk of depressive episodes or trigger new onset depression [15].

Reproductive psychiatry is primarily focused on women due to the unique psychological and physiological challenges they face during their reproductive years. Studies highlight the significant mental health burden in women with infertility, with one review of 44 studies reporting that 23% of women with infertility experience major depressive disorder (MDD), 13.3% generalized anxiety disorder (GAD), and 79% report experiencing acute stress [45]. Furthermore, psychopharmacological treatment in women during their reproductive years involves careful consideration of potential impacts on pregnancy, unlike in men, whose treatment does not usually carry these risks [39]. Psychopharmacological medications refer to the use of medications to treat and manage mental health disorders or psychological symptoms. These interventions aim to influence the functioning of the central nervous system, including the brain, to alleviate symptoms associated with various mental health conditions. There is ongoing controversy surrounding the use of psychopharmacology during infertility treatment, as concerns arise over the potential impact of these medications on fertility outcomes [10]. However, currently available studies found no psychopharmacological influence on ART outcomes [10, 20].

Attitudes towards psychopharmacology reveal a significant social divide, with some people displaying skepticism towards it. Research indicates that some individuals may view psychopharmacology as unnatural, and likely to worsen their conditions over time. They also perceive these medications as expensive, physically destabilizing, and weakening, leading to a preference for taking lower doses than prescribed [19]. A qualitative study conducted by Asher et al. [4] underlined the drawbacks connected to psychopharmacological treatments, such as experiencing side effects and withdrawal symptoms. On the other hand, with developing a patient's own pattern of use and forming more stable attitudes towards the treatment, the benefits of these interventions outweigh the previous concerns [4]. Improvements in public attitudes towards psychiatric treatment, including greater acceptance of medication, have been observed over the past twenty-five years [2]. Given the growing body of research in reproductive psychiatry, according to the Evidence-Based Research (EBR) approach, to prevent research waste, the first step in planning a new study should be conducting a systematic review (a synthesis of scientific data) to confirm the existence of a research gap. In this case, a scoping review was conducted to explore the psychopharmacotherapy among people with infertility undergoing ART procedures [31].

## Methods

The design and eligibility criteria for this project were established based on a pre-written protocol. We adhered to the protocol with two deviations: we did not limit our population to adults, as we decided to also include studies with people at risk of infertility (e.g., due to gender-affirming hormone treatment) who will use ART at a later stage, and we combined primary and current indications, as we believe this distinction was unnecessary. Additionally, the protocol was prospectively registered on the OSF (https://osf.io/egxw8/). This review followed the guidelines established by Tricco et al. [51] — Preferred Reporting Items for Systematic Reviews and Meta-Analyses for Scoping Reviews (PRISMA-ScR, Supplementary Material, List S1).

### Eligibility criteria

We formulated our research question according to the PCC framework (population, concept and context).

#### Population (P)

We considered including any participant experiencing infertility or at risk of infertility (in case of people before initiating gender-affirming hormone treatment). We excluded papers that solely focus on sexual dysfunction (such as disorders of orgasm and ejaculation) or gametes'parameters, unless these publications consider ART.

#### Concept (C)

We considered any psychotropic medications used according to Neuroscience-based Nomenclature (NbN) Classes (https://legacyfileshare.elsevier.com/promis_misc/NbN_Glossary.pdf). It is a system designed to provide a standardized and systematic naming convention for drugs that act on the nervous system. It was developed to overcome some of the limitations of traditional drug classification systems. Additionally, we considered including papers describing using drugs listed above in conditions not understood as psychiatric/psychological ones (known as “off-label” use) according to ICD/DSM or any other classification. Psychopharmacological medications could be prescribed by psychiatrists or other specialists.

#### Context (C)

We considered taking medication in relation to the period of ART as either before (anytime prior the ART procedures), during, or after ART (anytime). We adopt the U.S. Centers for Disease Control and Prevention (CDC) definition of ART which encompasses all fertility treatments involving the handling of eggs or embryos. Typically, ART procedures entail the surgical extraction of eggs from a woman's ovaries, their fusion with sperm in a laboratory setting, and subsequent implantation back into the woman's body or donation to another woman. Excluded from ART are treatments solely involving sperm handling, such as intrauterine (or artificial) insemination, and procedures where a woman takes medication solely to stimulate egg production without the aim of egg retrieval [57].

We considered any type of study. We used reviews to identify any relevant primary studies that were not identified in our searches.

### Searches

We systematically searched electronic databases (Ovid MEDLINE, Embase, CENTRAL, Web of Science, PsycInfo, Scopus) using adequate search strategies with no restrictions on the language and date, up to 15.02.2025. In developing the search strategy for MEDLINE, we combined the Medical Subject Headings (MeSH) and full-text words. In Supplementary Material, List S2, we present the used strategies. We manually searched references of reviews which come up with search to identify additional papers.

### Data selection and extraction

Using Endnote X8 (Clarivate Analytics ®) and Rayyan [38], we checked identified references for duplicates.

All steps (titles and abstracts screening, full-text assessments, and extraction) were done independently by two reviewers. Conflicts were resolved with discussion and help from the third reviewer. To ensure an adequate understanding of the criteria, we proceeded with calibration exercises before these steps. In case of missing data, we planned to request it from study authors.

The features extracted from the data encompassed the type of Assisted Reproductive Technology (ART) utilized, the reasons motivating ART usage, demographic information including age, sex, and presence of psychiatric diagnoses among the population, details regarding the type of medication administered, the indications for medication use, the timing of medication administration relative to ART (before, during, or after procedures), outcomes associated with ART including success rates, birth outcomes, and psychological impacts, as well as identification of existing gaps in research and suggestions for future studies to address these gaps and enhance understanding and outcomes in ART.

### Data synthesis

We used the aggregated data extracted from the trials. When combining the data was justified, we performed a descriptive analysis in Microsoft Excel. Studies that did not provide amenable data for quantitative analysis were assessed in a narrative summary.

### Analysis of subgroups

We performed analysis within the following subgroups: (1) group of medication according to NbN classes; (2) type of indication for using a specific medication (psychiatric/non-psychiatric).

## Results

### Results of the search

The database searches identified 7767 records. This resulted in 5631 records after removing duplicates. Based on titles and abstracts, we excluded 5555 irrelevant records, resulting in retrieving 76 records (left side of PRISMA Flowchart). Additionally, from 16 identified reviews (Supplementary Material, Table S6), we identified 27 primary studies - five of them met our eligibility criteria [1, 6, 9, 27, 46].

**Figure 1:**
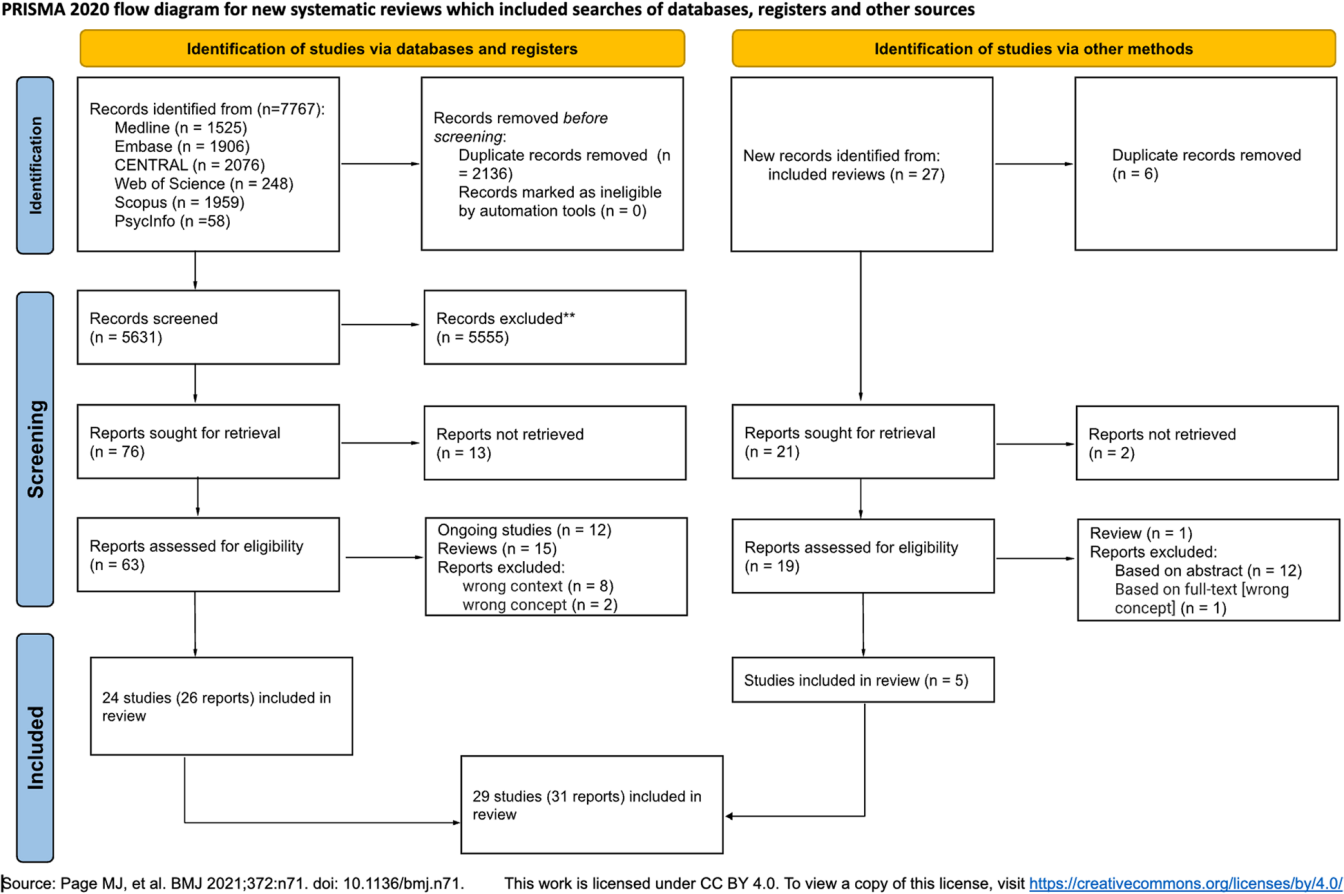
PRISMA flowchart

Altogether (after database and snowball searches), we included 29 studies published in 31 papers [1, 3, 6, 8–10, 13, 15, 16, 18, 20, 24–27, 30, 32, 33, 35, 37, 40, 41, 43, 44, 46, 47, 49, 52, 53]. The PRISMA flow diagram is presented in Figure 1.

In total, we could not retrieve 13 full texts of studies therefore we labeled them as awaiting classification (Supplementary Material, List S3) and they will be checked during each update of the search. We excluded 11 studies: eight due to the wrong context and three due to the wrong concept (Supplementary Material, List S4). We identified three protocols of studies and nine abstracts which we labeled as ongoing studies (Supplementary Material, List S5).

### Included studies

#### General characteristics

The general characteristics regarding study types, funding, conflict of interests, ethical approval, and consent from participants are presented in Supplementary Material, Table S7.

We included 29 studies which were published between 1999 [9] and 2023 [18]. Nine of the research were conducted in the US (32.14%), three in Iran (10.71%), two in each country: the UK, China, Sweden, Israel, Portugal, and Brazil (7.14%), and one in each: Italy, Turkey, Denmark, Japan, and the Netherlands (3.57%).

#### Population

The total number of participants was 746,659 (median 74; from 1 to 575,921) — 745,749 (99.88%) women and 910 (0.12%) men. Age of the participants was presented in a heterogeneous way making it difficult to synthesize, however, in all studies if reported, the people were in early 30 to 45 years. In two studies [28,43], the population involves centers of specialists. The characteristics of the participants are presented in Table 1.

**Table 1:** General characteristics of participants included in studies (N=29)

Variable	Studies	
	n	%
Type of ART		
IVF	22	75.86
ICSI	11	37.93
FET	2	6.90
egg donation	1	3.45
NR	2	6.90
Reason for ART		
general male factor	10	34.48
unexplained	8	27.59
tubal factor	6	20.69
general female factor	5	17.24
combined	5	17.24
endometriosis	5	17.24
infertility	5	17.24
ovulatory	4	13.79
PCOS	2	6.90
uterine	2	6.90
given the age	1	3.45
NR	12	41.38
Psychiatric diagnosis		
depression (MDD, minor)	12	41.38
unspecified anxiety disorders	8	27.59
dysthymia	2	6.90
GAD	2	6.90
bipolar disorder	2	6.90
unspecified eating disorder	2	6.90
OCD	2	6.90
panic disorder	2	6.90
adjustment disorder	1	3.45
psychotic episode	1	3.45
unspecified mood disorders	1	3.45
hypo-/mania	1	3.45
agoraphobia	1	3.45
social phobia	1	3.45
anorexia	1	3.45
bulimia nervosa	1	3.45
insomnia	1	3.45
suicidal ideation	1	3.45
ASD	0	0
ADHD	0	0
NA	2	6.90
NR	12	41.38

*ADHD* attention deficit hyperactivity disorder; *ART* Assisted reproductive technology; *ASD* autistic spectrum disorder; *FET* frozen embryo transfer; *GAD* generalized anxiety disorder; *ICSI* Intracytoplasmic Sperm Injection; *IVF* in vitro fertilization; *MDD* major depressive disorder; *NA* not applicable; *NR* not reported; *OCD* obsessive-compulsive disorder; *PCOS* polycystic ovary syndrome

All participants included in the reviewed studies had documented infertility. None of the studies focused on individuals merely at risk of infertility. One abstract (labelled as an ongoing study) included a sample of 26 transgender women, but no outcomes were yet reported.

#### Medication

Altogether, psychiatric medications occurred 83 times: 45 times (54.2%) medications for depression, 16 times (19.3%) - for anxiety, 9 times (10.8%) - for psychosis, 5 times (6%) mood stabilizers and 8 times (9.6%) other drugs. Regarding the time of use of the medication, most of them were used during ART (n=20/29, 68.97%), before ART (n=13/29, 44.83%), and after ART (n=7/29, 24.14%). In 3 papers (6.98%) this information was not provided.

##### Medications for depression

Serotonin reuptake inhibitor (SERT) constitutes the most frequently explored group of drugs for depression (n=15/29, 51.72%) with fluoxetine the most common medication (n=8/29, 27.59%). While, the second most frequently tested group was serotonin and norepinephrine reuptake inhibitors (SERT and NET) (n=4/29, 13.79%) with venlafaxine on the top (n=3/29, 10.34%) (Table 2).

**Table 2:** List of antidepressants examined in studies (N=29)

NbN glossary	Medications	Studies	
		n	%
SERT	SSRI (general)	4	13.79
	fluoxetine	8	27.59
	paroxetine	2	6.90
	sertraline	5	17.24
	escitalopram	3	10.34
	citalopram	3	10.34
	fluvoxamine	1	3.45
SERT and NET	SNRI (general)	1	3.45
	venlafaxine	3	10.34
	duloxetine	1	3.45
	amoxapine	1	3.45
	clomipramine	1	3.45
Melatonin, serotonin agonist and antagonist	agomelatine	1	3.45
Norepinephrine MM	mianserin	1	3.45
Histamine, norepinephrine, serotonin antagonist	mirtazapine	1	3.45
-	tricyclic (general)	1	3.45
MM; SERT and NET, receptor antagonist (5-HT2)	amitriptyline	1	3.45
NET, DAT, norepinephrine, dopamine releaser	bupropion	1	3.45
serotonin antagonist and agonist	nefazodone	1	3.45
-	not specified antidepressants	6	20.69

*DAT* dopamine reuptake inhibitor; *MM* (Multi-Model) = more than one mode of action; *NET* norepinephrine reuptake inhibitor; *SERT* Serotonin reuptake inhibitor; *SNRI* serotonin norepinephrine reuptake inhibitor; *SSRI* selective serotonin reuptake inhibitor

##### Medications for anxiety

The most frequently examined medications were GABA Positive Allosteric Modulators with midazolam the most commonly discussed (n=6/29, 20.69%). Within this group were also diazepam (n=3/29, 10.34%), temazepam (n=2/29, 6.90%), and oxazepam, clonazepam, lorazepam which were examined once (n=1/29, 3.45%). Additionally, the general ‘benzodiazepines’ and ‘anxiolytics’ were tested once too (n=1/29, 3.45%).

##### Medications for psychosis

There were several antipsychotics examined in primary studies: olanzapine (dopamine, serotonin receptor antagonist (D2, 5-HT2)), clozapine, risperidone (dopamine, serotonin, noradrenaline receptor antagonist (D2, 5-HT2, NE alpha-2)), quetiapine (dopamine, serotonin, noradrenaline MM; receptor antagonist (D2, 5-HT2) and reuptake inhibitor (NET)), aripiprazole (dopamine, serotonin receptor partial agonist (D2, 5-HT1 A)), haloperidol (dopamine receptor antagonist (D2)), and promethazine (histamine, dopamine receptor antagonist (H1,D2)). None of the medicine was investigated more than once.

##### Medications for relapse prevention

Among medications that could be used in bipolar disorder, there were: valproic acid (acting on glutamate in unknown yet mode of action), lithium (acting on enzyme interactions), lamotrigine (acting on glutamate via voltage-gated sodium channel blocker), and general mood stabilizers.

##### Others

The remaining medications involved medication in sleep disturbances, opioid use disorder, or toxicology: zolpidem (GABA Positive Allosteric Modulator (GABA-A receptor, benzodiazepine site)), flumazenil (GABA receptor antagonist), ketamine (glutamate receptor antagonist), naloxone (opioid receptor antagonist), methadone (opioid receptor agonist), and other (hypnotics, not specified sedatives).

#### The indication for using a specific medication

A) Psychiatric indications are presented in Table 3.

**Table 3:** Map of psychiatric indications of medications

	Dosages/time	psychotic symptoms	anxiety	depression	insomnia	bipolar disorder	delirium
sedatives	NR/NR				Goisis, 2023 [18]		
hipnotics	NR/NR				Goisis, 2023 [18]		
zolpidem	NR/NR				McIntosh, 2010 [33]		
lamotrigine	NR/NR					Freeman, 2016[15]	
lithium	NR/NR					Siegel, 2022 [47] Freeman, 2016 [15]	
valproic acid	NR/NR					Freeman, 2016 [15]	
haloperidol	0,5 mg x 3/1 week						Mercan, 2005 [35]
atypical antipsychotics	NR/NR					Freeman, 2016 [15]	
aripiprazole	25 mg d/NR	Rodrigues, 2014 [43]					
olanzapine	NR/NR					Siegel, 2022 [47]	
risperidone	NR/NR					Siegel, 2022 [47]	
quetiapine	NR/NR					Siegel, 2022 [47]	
clozapine	NR/NR					Siegel, 2022 [47]	
antidepressants	NR/NR			Goisis, 2023 [18]			
SSRI	NR/at least 1 month before and throughout the patient's IVF and continued 12 to 14 weeks'gestation		Hernandez-Nieto, 2017 [20]	Hernandez-Nieto, 2017 [20]		Siegel, 2022 [47]	
sertraline	10–50 mg/at least 6 m		, 2016 [10]	Cesta, 2016 [10]			
fluoxetine	20 mg (5 studies) to 60 mg (1 study)/from 26 d to several years ^a^		Cesta, 2016 [10] Serafini, 2009 [46]	Cesta, 2016 [10] Keshavarz, 2020 [25] Keshavarz, 2021 [26] Ramezanzadeh, 2011[41] Applegarth, 2015 [3]			
citalopram	NR/at least 6 m		Cesta, 2016 [10]	Cesta, 2016 [10]			
paroxetine	NR/at least 6 m		Cesta, 2016 [10]	Cesta, 2016 [10]			
fluvoxamine	NR/at least 6 m		Cesta, 2016 [10]	Cesta, 2016 [10]			
escitalopram	5 mg d for a week, then 10 mg/at least 30 days before the first day of the menstrual cycle; 4 weeks during IVF cycle, total — 8 weeks or at least 6 m		Cesta, 2016 [10] Aisenberg Romano, 2019 [1]	Cesta, 2016 [10] Aisenberg Romano, 2019 [1]			
venlafaxine	NR/at least 6 m		Cesta, 2016 [10]	Cesta, 2016 [10] McIntosh, 2010 [33]			
clomipramine	NR/at least 6 m		Cesta, 2016 [10]	Cesta, 2016 [10]			
amitriptyline	NR/at least 6 m		Cesta, 2016 [10]	Cesta, 2016 [10]			
mianserin	NR/at least 6 m		Cesta, 2016 [10]	Cesta, 2016 [10]			
mirtazapine	NR/at least 6 m		Cesta, 2016 [10]	Cesta, 2016 [10]			
duloxetine	NR/at least 6 m		Cesta, 2016 [10]	Cesta, 2016 [10]			
bupropion	NR/at least 6 m		Cesta, 2016 [10]	Cesta, 2016 [10]			
agomelatine	NR/at least 6 m		Cesta, 2016 [10]	Cesta, 2016 [10]			
anxiolytics	NR/NR		Goisis, 2023 [18]				
diazepam	10–20 mg/NR		McIntosh, 2010 [33] Roest, 2019 [44]				
clonazepam	0.5 mg 2x day/3 w		Rodrigues, 2014 [43]				
Lorazepam	1 mg/NR		Roest, 2019 [44]				
Midazolam	7.5 mg p.o.; 2–5 mg i.v.; 5 mg i.m./NR		Roest, 2019 [44]				
Temazepam	10 mg/NR		Roest, 2019 [44]				
Oxazepam	10–20 mg/NR		Roest, 2019 [44]				

*NR* not reported; *w* week; *d* day; *m* month; *p.o.* per os; *i.v*. intravenous; *i.m.* intramuscular injection

^a^additionally cognitive-behavioral therapy was conducted

+ represents reporting in a single study

In 7 full texts, the indications were not reported. It regards to use of antidepressants (in 4 cases); fluoxetine, sertraline, and citalopram (each twice); and escitalopram; paroxetine; venlafaxine; nefazodone, SSRI (once).

b) Non-psychiatric indications of psychopharmacology are presented in Table 4.

**Table 4:** Map of non-psychiatric indications of medications described in studies

	Dosages/time	sedation	emergency	ejaculation failure	analgesia	anaesthesia
promethazine	5 mg/NR	Ng, 2002 [37]				
sertraline	NR/NR			Lu, 2009 [30]		
amoxapine	NR/NR			Karibe, 2024 [24]		
diazepam	1–10 mg, i.v. or 10–20 mg p.o./NR	Elkington, 2003 [13] Ng, 2002 [37]				
midazolam	5–10 mg, i.v.; 0.05–0.06 mg/kg infusion/NR	Ben-Shlomo, 1999 [6] Elkington, 2003 [13]				Casati, 1999 [9]
temazepam	20 mg p.o./NR	Elkington, 2003 [13]				
naloxone	NR/NR		Elkington, 2003 [13]			
flumazenil	NR//NR		Elkington, 2003 [13]			
methadone	5–7.5 mg,i.m/NR				Roest, 2019 [44]	
ketamine	0.75 mg per kg/NR	Ben-Shlomo, 1999 [6]				

*NR* not reported; *p.o.* per os; *i.v.* intravenous; *i.m.* intramuscular injection

#### Dosages of medications

The reported dosages together with the duration of treatment are presented in Tables 3 and 4. Dosages were not reported 43 times: antidepressants (n=6), SSRI (n=3), citalopram (n=2), fluoxetine (n=2), lithium (n=2), venlafaxine (n=2), sertraline (n=2), anxiolytics (n=1), antipsychotics (n=1), sedatives (n=1), mood stabilizers (n=1), escitalopram (n=1), nefazodone (n=1), paroxetine (n=1), SNRI (n=1), tricyclic (n=1), benzodiazepines (n=1), atypical antipsychotics (n=1), clozapine (n=1), olanzapine (n=1), quetiapine (n=1), risperidone (n=1), lamotrigine (n=1), valproic acid (n=1), flumazenil (n=1), hipnotics (n=1), naloxone (n=1), zolpidem (n=1).

#### Outcomes

The outcomes analyzed in certain studies are presented in Supplementary Material, Table S8.

##### Related to ART

 Semen: In 26 studies semen outcomes were not considered. In the two remaining studies among outcomes were: initial total motile sperm count and final total motile sperm count — twice examined, initial total volume (ml), initial concentration (M/ml), initial motility (%), final total volume (ml), final concentration (M/ml), and final motility (%) tested once.

 Cycle: In six studies number of oocytes was reported.

 Efficacy: fertilization rate/pregnancy/positive conception was examined most frequently (10 times), and then number of embryos biopsied (3 times), and implantation rate (twice). Among single outcomes were: blastulation rate, number of usable blastocysts, number of euploid embryos, cycle cancellation rate, top quality embryos presence, and percentage of zygotes developing to 8-cell embryos,

Other: number of follicles punctured, transvaginal ultrasound-guided oocyte retrieval duration, number of harvested oocytes, number of initiated ART, day of transfer, day 13 hCG value, day 15 hCG value.

##### Related to birth

Live birth rate was examined in six studies. Multiple pregnancy rates were tested in one paper. Additionally, a spontaneous abortion rate was examined three times, together with early pregnancy loss, and miscarriage.

##### Related to mental state

Among outcomes related to mental states the most common one was severity of symptoms (psychotic, anxious, and depressive). The other outcomes include: sedation level, satisfaction level, TNF-α levels, cortisol levels, relapse rate, inflammatory biomarker predictors of relapse, relation between stress, incidence of side effects, time taken to achieve Aldrete score=10, and oxygen saturation.

## Discussion

### Summary of main results

This study sought to collect evidence regarding psychopharmacotherapy among people undergoing ART. The studies included in this review span over two decades, with the majority receiving non-industrial funding. They encompass a variety of research designs and involve nearly 750,000 participants, predominantly women (99.9%) aged between 30 and 45 years. The total number of participants is largely inflated due to the inclusion of large registry studies, which significantly increases the overall count. In contrast, the median sample size of 74 indicates that the majority of the studies had relatively small participant groups, suggesting that smaller-scale research is more common in this field. The most frequently studied fertility treatments were IVF and Intracytoplasmic Sperm Injection (ICSI), which were used for various infertility reasons. Psychopharmacology was primarily administered for the treatment of depression and anxiety disorders. Among this, serotonin reuptake inhibitors (SERT) were the most commonly studied class of drugs, with fluoxetine being the most frequently used medication. Additionally, psychopharmacology was also employed beyond typical psychiatric indications, most notably for sedating patients. The psychiatric indications were generally described in broad terms, and the medications used were typically first or second-line treatments. These drugs were often administered at lower-than-standard doses [5, 22, 36]. The most commonly reported endpoints in the studies involved the efficacy of ART procedures and the severity of psychiatric symptoms.

### Overall completeness, applicability of evidence, and identified gaps

While the studies cover a large sample size and diverse geographic regions, the focus on predominantly women participants limits the generalizability of the results to other populations, such as men or non-binary individuals (gap #1). The existing literature on psychopharmacotherapy in individuals undergoing ART is characterized by several important limitations. Psychiatric indications were frequently reported in broad or nonspecific terms (e.g.,"anxiety"without diagnostic clarification), and information on medication dosing was often absent (gap #2). This lack of clinical granularity limits the applicability of findings, particularly given the nuanced dosing considerations and off-label use that are often required in psychopharmacological practice.

Moreover, there was substantial variability in study designs, outcome measures, and reporting standards (gap #3), which complicates comparisons across studies and limits the generalizability of findings. A large proportion of the included studies were observational, and only approximately one-quarter were randomized controlled trials—most of which compared pharmacological interventions with placebo rather than active controls (gap #4). These design-related limitations, along with the risk of potential confounding in observational data, underscore the need for more rigorous and standardized research to inform evidence-based treatment recommendations in this population.

The lack of reporting of the features ranged from 29% to 54% (for general characteristics), from 7% to 43% - in case of the description of the population, and 7% for the medication section.

Substance use disorder (SUD), which was not analyzed in papers included in our review, is another prevalent issue in the general population [50] (gap #5). Epidemiological data suggest that SUD during pregnancy remains a significant concern, with varying prevalence depending on the type of substance and the population. Among those seeking ART, the prevalence of SUD might be underreported, partly due to the stigma and fear of legal or medical repercussions, which can lead to hesitancy in disclosing substance use [17].

Additionally, we observed that medications are often administered at lower-than-standard doses, with some dosages falling significantly below the minimum clinically effective level (gap #6). This cautious approach may be primarily driven by concerns about the potential teratogenic effects and other risks these drugs may pose to the developing fetus [54], or generally better tolerance and belief of the lower risk of side effects [11, 12, 23].

Nevertheless, several currently widely used medications were not discussed in primary studies, such as vortioxetine, trazodone, buspirone (classified as Category B in pregnancy by FDA, meaning animal studies have failed to show any risks associated with its use during pregnancy [55]), and long-acting injectable (LAI) forms of antipsychotics (gap #7).

Despite the inclusion of fertility preservation in numerous studies and guidelines for patients with cancer [34, 42], we did not identify any studies focusing on the use of psychopharmacology among this population in the context of ART (gap #8). The absence of robust research leaves a gap in knowledge regarding the safety, efficacy, and appropriate dosing of psychiatric medications for cancer survivors undergoing ART. This gap is particularly concerning given the potential interactions between cancer treatments, fertility preservation methods, and psychopharmacology.

### Implications for research

There is a clear need for more qualitative research to explore the personal experiences, emotions, and decision-making processes of individuals undergoing ART while using psychopharmacology. Qualitative studies could provide deeper insights into how mental health and fertility treatments intersect, helping to identify patient needs, preferences, and challenges that are often not captured in quantitative research. Additionally, large-scale survey-based studies could gather comprehensive data on the prevalence, attitudes, and outcomes related to psychopharmacotherapy among diverse populations undergoing ART, including men, non-binary individuals, and cancer survivors.

### Potential biases in the review process

Our definition of ART may be too narrow, potentially excluding relevant studies. By only considering certain types of ART (e.g., in vitro fertilization), we might overlook studies that explore psychopharmacotherapy in broader reproductive treatments, such as intrauterine insemination or ovarian stimulation without IVF. Additionally, by using the NbN classification system, we may exclude studies that examined psychopharmacology outside of this framework, even when the medication was used for psychiatric indications.

## Conclusions

There is generally little evidence on psychopharmacotherapy among individuals undergoing ART. Most of it includes medication for depression and anxiety disorders. Psychiatric medications may sometimes be used for non-psychiatric purposes. The reporting in studies is mostly poor.

## Supplementary Information

Supplementary Material 1

## Data Availability

No datasets were generated or analysed during the current study.

## References

[1] Aisenberg Romano, Gabi, et al. Prophylactic SSRI treatment for women suffering from mood and anxiety symptoms undergoing in vitro fertilization—a prospective placebo-controlled study. Archives of Women's Mental Health, 2019, 22: 503-510. 10.1007/s00737-018-0911-5 30225529

[2] Angermeyer, Matthias C., et al. Public attitudes towards psychiatry and psychiatric treatment at the beginning of the 21st century: a systematic review and meta‐analysis of population surveys. World Psychiatry, 2017, 16.1: 50-61. 10.1002/wps.20383 PMC5269489 28127931

[3] Applegarth, Linda D. Fertility counseling for individuals. Fertility Counseling. Cambridge, 2015.

[4] Asher, Maia; Roe, David; Hasson-Ohayon, Ilanit. Attitudes toward and patterns of medication use among people with serious mental illness: There’s more than meets the eye. Frontiers in Psychiatry, 2023, 14: 1133140. 10.3389/fpsyt.2023.1133140 PMC9983815 36873214

[5] Bandelow, Borwin, et al. World Federation of Societies of Biological Psychiatry (WFSBP) guidelines for treatment of anxiety, obsessive-compulsive and posttraumatic stress disorders–Version 3. Part I: Anxiety disorders. The World Journal of Biological Psychiatry, 2023, 24.2: 79-117. 10.1080/15622975.2022.2086295 35900161

[6] Ben-Shlomo, Izhar, et al. Midazolam/ketamine sedative combination compared with fentanyl/propofol/isoflurane anaesthesia for oocyte retrieval. Human Reproduction, 1999, 14.7: 1757-1759. 10.1093/humrep/14.7.1757 10402383

[7] Boivin, Jacky; Griffiths, Emily; Venetis, Christos A. Emotional distress in infertile women and failure of assisted reproductive technologies: meta-analysis of prospective psychosocial studies. BmJ, 2011, 342. 10.1136/bmj.d223 PMC3043530 21345903

[8] Carvalho, Claudia F., et al. Psychotropic medication use among women seeking assisted reproductive technology (ART) therapy: a cross-sectional study. Journal of Affective Disorders, 2021, 292: 386-390. 10.1016/j.jad.2021.05.063 34139412

[9] Casati A, et al. Anaesthesia for ultrasound guided oocyte retrieval: midazolam/remifentanil versus propofol/fentanyl regimens. European journal of anaesthesiology. 1999;16(11):773–8. 10713871 10.1046/j.1365-2346.1999.00584.x

[10] Cesta, Carolyn E., et al. Depression, anxiety, and antidepressant treatment in women: association with in vitro fertilization outcome. Fertility and sterility, 2016a, 105.6: 1594-1602. e3. 10.1016/j.fertnstert.2016.01.036 26920258

[11] Daughton Christian G, Ruhoy Ilene Sue. Lower-dose prescribing: minimizing “side effects” of pharmaceuticals on society and the environment. Science of the total Environment. 2013;443:324–37. 23201698 10.1016/j.scitotenv.2012.10.092

[12] De Jong, Veronica; Raz, Amir. Sub-Therapeutic doses in the treatment of depression: the implications of Starting low and going Slow. The Journal of Mind-Body Regulation, 2011, 1.2: 73-84.

[13] Elkington, Nicholas M.; KEHOE, Jane; Acharya, Umesh. Intravenous sedation in assisted conception units: a UK survey. Human Fertility, 2003, 6.2: 74-76. 10.1080/1464770312331369083 12869788

[14] Farquhar, Cindy; Marjoribanks, Jane. Assisted reproductive technology: an overview of Cochrane Reviews. Cochrane Database of Systematic Reviews, 2018, 8. 10.1002/14651858.CD010537.pub5 PMC6953328 30117155

[15] Freeman, Marlene P., et al. Predictors of depressive relapse in women undergoing infertility treatment. Journal of women's health, 2018, 27.11: 1408-1414. 10.1089/jwh.2017.6878 PMC6247370 30067141

[16] Friedman, Brooke E., et al. Effect of selective serotonin reuptake inhibitors on in vitro fertilization outcome. Fertility and sterility, 2009, 92.4: 1312-1314. 10.1016/j.fertnstert.2009.03.060 19423105

[17] Frew, Julia R. Reducing the stigma of perinatal substance use disorders: The time is now. Archives of Women's Mental Health, 2023, 26.3: 411-413. 10.1007/s00737-023-01322-3 37103582

[18] Goisis, Alice, et al. Medically assisted reproduction and mental health: a 24-year longitudinal analysis using Finnish register data. American journal of obstetrics and gynecology, 2023, 228.3: 311. e1-311. e24. 10.1016/j.ajog.2022.10.041 36336083

[19] Grover, Sandeep, et al. Attitude toward psychotropic medications: A comparison of the elderly and adult patients with affective and psychotic disorders. Journal of Geriatric Mental Health, 2019, 6.2: 38-45.

[20] Hernandez-Nieto, Carlos, et al. Embryo aneuploidy is not impacted by selective serotonin reuptake inhibitor exposure. Fertility and Sterility, 2017a, 108.6: 973-979. 10.1016/j.fertnstert.2017.08.040 29202974

[21] Jain, Meaghan; Singh, Manvinder. Assisted reproductive technology (ART) techniques. 2022. 35015434

[22] Johnson, Chris F. Depression treatment, for adults, in primary care. 2024. https://rightdecisions.scot.nhs.uk/media/ykxn2psm/v6-36-depression-treatment-for-adults-in-primary-care.pdf (accessed 3.04.2025)

[23] Kampermann, Lea; Nestoriuc, Yvonne; Shedden-Mora, Meike C. Physicians’ beliefs about placebo and nocebo effects in antidepressants–an online survey among German practitioners. PLoS One, 2017, 12.5: e0178719. 10.1371/journal.pone.0178719 PMC5451122 28562635

[24] Karibe, Jurii, et al. Clinical case of 45, X/46, XY mosaic male with ejaculatory disorder associated with seminal vesicle dysplasia: a case report. Sexual Medicine, 2024, 12.5: qfae066. 10.1093/sexmed/qfae066 PMC11443022 39360231

[25] Keshavarz, Sedigheh, et al. The effect of antidepressant treatment on the HPA axis, changes in depression score and serum levels of TNF-α in depressed infertile women. Archives of Clinical Psychiatry (São Paulo), 2020, 47.1: 7-12.

[26] Keshavarz, Sedigheh, et al. Comparison of Midwifery Consultation and Fluoxetine on IVF Outcomes in Depressed Infertile Women: A Clinical Trial Research Study. Current Women's Health Reviews, 2021, 17.2: 176-184.

[27] Klock, Susan C., et al. A pilot study of the relationship between selective serotonin reuptake inhibitors and in vitro fertilization outcome. Fertility and sterility, 2004, 82.4: 968-969. 10.1016/j.fertnstert.2004.02.139 15482784

[28] Liang, Yuanhao, et al. Global, regional, and national prevalence and trends of infertility among individuals of reproductive age (15–49 years) from 1990 to 2021, with projections to 2040. Human Reproduction, 2025, deae292. 10.1093/humrep/deae292 39752330

[29] Liu, Yao-Fang, et al. The analysis of anxiety and depression in different stages of in vitro fertilization-embryo transfer in couples in China. Neuropsychiatric disease and treatment, 2021, 649-657. 10.2147/NDT.S287198 PMC7920591 33658786

[30] Lu, Shaoming, et al. Combined use of phosphodiesterase-5 inhibitors and selective serotonin reuptake inhibitors for temporary ejaculation failure in couple undergoing assisted reproductive technologies. Fertility and sterility, 2009, 91.5: 1806-1808. 10.1016/j.fertnstert.2008.03.003 18440512

[31] Lund, Hans, et al. How to improve the study design of clinical trials in internal medicine: Recent advances in the evidence-based methodology. 2021. 10.20452/pamw.16076 34590450

[32] Malling, GM Hviid, et al. The association between antidepressant use and assisted reproductive technology (ART) treatment in Danish women: A national registry-based cohort study. European Journal of Obstetrics & Gynecology and Reproductive Biology, 2021, 258: 401-408. 10.1016/j.ejogrb.2020.12.019 33550215

[33] Mcintosh Michael D, Ferrando Stephen. Perimenopausal postpartum depression after conception by assisted reproductive technology. Psychosomatics. 2010;51(4):345–8. 20587765 10.1176/appi.psy.51.4.345

[34] Meernik, Clare, et al. Fertility preservation and financial hardship among adolescent and young adult women with cancer. Cancer Epidemiology, Biomarkers & Prevention, 2022, 31.5: 1043-1051. 10.1158/1055-9965.EPI-21-1305 PMC9074091 35506248

[35] Mercan Sibel, Mercan Ramazan, Karamustafalioglu Oguz. Case report: delirium associated with ovarian hyperstimulation syndrome. Reproductive biomedicine online. 2005;10(2):178–81. 15823220 10.1016/s1472-6483(10)60938-8

[36] National Institute for Health and Care Excellence (NICE). Depression in adults: treatment and management. NICE guideline (NG222). June 29, 2022 35977056

[37] Ng, Ernest Hung Yu; MIAO, Benyu; HO, Pak Chung. Anxiolytic premedication reduces preoperative anxiety and pain during oocyte retrieval. A randomized double-blinded placebo-controlled trial. Human Reproduction, 2002, 17.5: 1233-1238. 10.1093/humrep/17.5.1233 11980744

[38] Ouzzani, Mourad, et al. Rayyan—a web and mobile app for systematic reviews. Systematic reviews, 2016, 5: 1-10. 10.1186/s13643-016-0384-4 PMC5139140 27919275

[39] Payne, Jennifer L. Reproductive psychiatry: giving birth to a new subspecialty. International Review of Psychiatry, 2019, 31.3: 207-209. 10.1080/09540261.2018.1579991 31241010

[40] Pedro, Juliana, et al. Infertility-related stress and the risk of antidepressants prescription in women: a 10-year register study. Human Reproduction, 2019, 34.8: 1505-1513. 10.1093/humrep/dez110 31339996

[41] Ramezanzadeh, Fatemeh, et al. Psychiatric intervention improved pregnancy rates in infertile couples. the malaysian journal of medical sciences: MJMS, 2011, 18.1: 16. PMC3216204 22135569

[42] Rives, Nathalie, et al. What should be done in terms of fertility preservation for patients with cancer? The French 2021 guidelines. Eur J Cancer, 2022, 173: 146-166 10.1016/j.ejca.2022.05.013 35932626

[43] Rodrigues, José Daniel Machado; LAPA, Maria Georgina Santos; BROCKINGTON, Ian Fraser. Psychotic episode secondary to gonadotrophins. General Hospital Psychiatry, 2014, 36.5: 549. e7-549. e8. 10.1016/j.genhosppsych.2014.05.016 24996859

[44] Roest, Inez, et al. Different methods of pain relief for IVF and ICSI oocyte retrieval–A Dutch survey. European Journal of Obstetrics & Gynecology and Reproductive Biology: X, 2019, 4: 100065. 10.1016/j.eurox.2019.100065 PMC6728718 31517299

[45] Salari, Nader, et al. Global prevalence of major depressive disorder, generalized anxiety, stress, and depression among infertile women: a systematic review and meta-analysis. Archives of Gynecology and Obstetrics, 2024, 309.5: 1833-1846. 10.1007/s00404-024-07444-y 38459997

[46] Serafini, Paulo, et al. Fluoxetine treatment for anxiety in women undergoing in vitro fertilization. International Journal of Gynecology & Obstetrics, 2009, 105.2: 136-139. 10.1016/j.ijgo.2008.12.013 19201400

[47] Siegel Andrew M, Ravitsky Vardit. Women with mental illness seeking assisted reproduction considerations in ethical candidate selection. Current Psychiatry Reports. 2018;20:1–8. 30094584 10.1007/s11920-018-0944-5

[48] Sun, Hui, et al. Global, regional, and national prevalence and disability-adjusted life-years for infertility in 195 countries and territories, 1990–2017: results from a global burden of disease study, 2017. Aging (Albany NY), 2019, 11.23: 10952. 10.18632/aging.102497 PMC6932903 31790362

[49] Sylvester, Christie, et al. Disordered eating and distress in women seeking in vitro fertilization. Journal of Psychosomatic Research, 2020, 128: 109880. 10.1016/j.jpsychores.2019.109880 31760342

[50] Ten Have, Margreet, et al. Prevalence and trends of common mental disorders from 2007‐2009 to 2019‐2022: results from the Netherlands Mental Health Survey and Incidence Studies (NEMESIS), including comparison of prevalence rates before vs. during the COVID‐19 pandemic. World Psychiatry, 2023, 22.2: 275-285. 10.1002/wps.21087 PMC10168151 37159351

[51] Tricco, Andrea C., et al. PRISMA extension for scoping reviews (PRISMA-ScR): checklist and explanation. Annals of internal medicine, 2018, 169.7: 467-473. 10.7326/M18-0850 30178033

[52] VOLGSTEN, Helena, et al. Prevalence of psychiatric disorders in infertile women and men undergoing in vitro fertilization treatment. Human Reproduction, 2008, 23.9: 2056-2063. 10.1093/humrep/den154 PMC2517152 18583334

[53] Walker, Zachary, et al. The effects of male anxiety and depression on IVF outcomes. Human Reproduction, 2023, 38.11: 2119-2127. 10.1093/humrep/dead179 37690112

[54] Westin, Andreas Austgulen; Reimers, Arne; Spigset, Olav. Should pregnant women receive lower or higher medication doses?. Tidsskrift for Den norske legeforening, 2018. 10.4045/tidsskr.18.0065 30378417

[55] Wilson, T. K.; Tripp, J. Buspirone. StatPearls. Treasure. 2024.

[56] World Health Organization. Infertility prevalence estimates, 1990–2021. World Health Organization, 2023. https://www.who.int/publications/i/item/978920068315

[57] Zhang, Li, et al. Prevalence and associated risk factors for anxiety and depression in infertile couples of ART treatment: a cross-sectional study. BMC psychiatry, 2022, 22.1: 616. 10.1186/s12888-022-04256-9 PMC9483863 36123644

[58] Zheng, Ju-Fen, et al. ICSI treatment of severe male infertility can achieve prospective embryo quality compared with IVF of fertile donor sperm on sibling oocytes. Asian Journal of Andrology, 2015, 17.5: 845-849. 10.4103/1008-682X.146971 PMC4577602 25652630

