# Supplementary material for: Psychopharmacological interventions among people who use Assisted Reproductive Technology (ART) — a scoping review
Source: Reprod Biol Endocrinol. 2025 May 10;23:65. doi: 10.1186/s12958-025-01400-4 (PMC12065253; doi:10.1186/s12958-025-01400-4)
Supplement: Supplementary file 1 — Supplementary Material 1 [file 12958_2025_1400_MOESM1_ESM.docx]

Supplementary Material, List S1 PRISMA-ScR Checklist

**Preferred Reporting Items for Systematic reviews and Meta-Analyses extension for Scoping Reviews (PRISMA-ScR) Checklist**

| **SECTION** | **ITEM** | **PRISMA-ScR CHECKLIST ITEM** | **REPORTED ON PAGE #** |
| --- | --- | --- | --- |
| **TITLE** | | | |
| Title | 1 | Identify the report as a scoping review. | 1 |
| **ABSTRACT** | | | |
| Structured summary | 2 | Provide a structured summary that includes (as applicable): background, objectives, eligibility criteria, sources of evidence, charting methods, results, and conclusions that relate to the review questions and objectives. | 2,3 |
| **INTRODUCTION** | | | |
| Rationale | 3 | Describe the rationale for the review in the context of what is already known. Explain why the review questions/objectives lend themselves to a scoping review approach. | 4-6 |
| Objectives | 4 | Provide an explicit statement of the questions and objectives being addressed with reference to their key elements (e.g., population or participants, concepts, and context) or other relevant key elements used to conceptualize the review questions and/or objectives. | 6 |
| **METHODS** | | | |
| Protocol and registration | 5 | Indicate whether a review protocol exists; state if and where it can be accessed (e.g., a Web address); and if available, provide registration information, including the registration number. | 6 |
| Eligibility criteria | 6 | Specify characteristics of the sources of evidence used as eligibility criteria (e.g., years considered, language, and publication status), and provide a rationale. | 7,8 |
| Information sources* | 7 | Describe all information sources in the search (e.g., databases with dates of coverage and contact with authors to identify additional sources), as well as the date the most recent search was executed. | 8 |
| Search | 8 | Present the full electronic search strategy for at least 1 database, including any limits used, such that it could be repeated. | Supplementary Material List S2 |
| Selection of sources of evidence | 9 | State the process for selecting sources of evidence (i.e., screening and eligibility) included in the scoping review. | 8,9 |
| Data charting process | 10 | Describe the methods of charting data from the included sources of evidence (e.g., calibrated forms or forms that have been tested by the team before their use, and whether data charting was done independently or in duplicate) and any processes for obtaining and confirming data from investigators. | 8,9 |
| Data items | 11 | List and define all variables for which data were sought and any assumptions and simplifications made. | 9 |
| Critical appraisal of individual sources of evidence | 12 | If done, provide a rationale for conducting a critical appraisal of included sources of evidence; describe the methods used and how this information was used in any data synthesis (if appropriate). | NA |
| Synthesis of results | 13 | Describe the methods of handling and summarizing the data that were charted. | 9 |
| **RESULTS** | | | |
| Selection of sources of evidence | 14 | Give numbers of sources of evidence screened, assessed for eligibility, and included in the review, with reasons for exclusions at each stage, ideally using a flow diagram. | 10, Fig 1 |
| Characteristics of sources of evidence | 15 | For each source of evidence, present characteristics for which data were charted and provide the citations. | 11-16, Table 1-4 |
| Critical appraisal within sources of evidence | 16 | If done, present data on critical appraisal of included sources of evidence (see item 12). | NA |
| Results of individual sources of evidence | 17 | For each included source of evidence, present the relevant data that were charted that relate to the review questions and objectives. | 11-16, Table 1-4 |
| Synthesis of results | 18 | Summarize and/or present the charting results as they relate to the review questions and objectives. | 11-16, Table 1-4 |
| **DISCUSSION** | | | |
| Summary of evidence | 19 | Summarize the main results (including an overview of concepts, themes, and types of evidence available), link to the review questions and objectives, and consider the relevance to key groups. | 16,17 |
| Limitations | 20 | Discuss the limitations of the scoping review process. | 19 |
| Conclusions | 21 | Provide a general interpretation of the results with respect to the review questions and objectives, as well as potential implications and/or next steps. | 20 |
| **FUNDING** | | | |
| Funding | 22 | Describe sources of funding for the included sources of evidence, as well as sources of funding for the scoping review. Describe the role of the funders of the scoping review. | 20 |

JBI = Joanna Briggs Institute; PRISMA-ScR = Preferred Reporting Items for Systematic reviews and Meta-Analyses extension for Scoping Reviews.

*From:* Tricco AC, Lillie E, Zarin W, O'Brien KK, Colquhoun H, Levac D, et al. PRISMA Extension for Scoping Reviews (PRISMAScR): Checklist and Explanation. Ann Intern Med. 2018;169:467–473. [doi: 10.7326/M18-0850](http://annals.org/aim/fullarticle/2700389/prisma-extension-scoping-reviews-prisma-scr-checklist-explanation).

Supplementary Material, List S2 Search strategies

**Medline**

exp embryo transfer/ or exp fertilization in vitro/ or exp sperm injections, intracytoplasmic/ or exp zygote intrafallopian transfer/ OR exp reproductive techniques, assisted/ or exp insemination, artificial/ or exp ovulation induction/ OR exp insemination, artificial/ or exp insemination, artificial, homologous/ OR exp Clomiphene/ OR exp oocyte donation/

(in Vitro adj2 fertili*).tw. OR (ivf or icsi or ZIFT).tw. OR (intracytopla* adj2 sperm).tw. OR zygote intrafallopian transfer*.tw. OR (embryo transfer* or ET).tw. OR (blastocyst adj2 transfer*).tw. OR assisted reproduct*.tw. OR artificial inseminat*.tw. OR iui.tw. OR intrauterine insemination*.tw. OR ovulation induc*.tw. OR (ovari* adj2 stimulat*).tw. OR superovulat*.tw. OR ovarian hyperstimulation.tw. OR COH.tw. OR (ovari* adj2 induction).tw. OR “intra‐uterine” insemination*.tw. OR clomi*.tw. OR ART.tw. OR ((want or wanting or hoping or hope or plan or planning or intend* or intention or contemplat*) adj5 pregnan*).tw.

exp Infertility/ OR exp male infertility/ OR exp Spermatozoa/ OR exp Infertility, Female/

(asthenozoospermi* or oligospermi* or azoospermi*).tw. OR Asthenospermia.tw. OR Teratospermia.tw. OR subfertil*.tw. OR infertil*.tw. OR oligoasthenoteratozoospermi*.tw. OR Oligozoospermi*.tw. OR Aspermi*.tw. OR Sperm*.tw. OR semen.tw.

exp Narcotics/ OR exp Analgesics, Opioid/ OR exp Psychotropic Drugs/ OR exp Central Nervous System Stimulants/ OR exp Amphetamines/ OR exp Hypnotics and Sedatives/ OR exp Benzodiazepines/ OR exp Morphine Derivatives/ OR exp Cocaine/ OR exp Methamphetamine/ OR exp Testosterone Congeners/ OR exp Psychopharmacology/ OR exp Antidepressive Agents/ OR exp Antimanic Agents/ OR exp Antipsychotic Agents/

((medication* OR pharmacotherapy OR treatment) AND antidepressant*).tw. OR (selective serotonin reuptake inhibitor*.tw. OR exp Serotonin Uptake Inhibitors/ OR SSRIs.tw. OR (serotonin AND norepinephrine reuptake inhibitor*).tw. OR SNRIs.tw. OR (norepinephrine AND dopamine reuptake inhibitors).tw. OR NDRIs.tw. OR Tricyclic antidepressant*.tw. OR TCAs.tw. OR monoamine oxidase inhibitor*.tw. OR MAOIs.tw.) AND (network.tw. OR “mixed-treatment”.tw. OR multiple treatment*.tw.) OR (buspirone.tw. OR phenelzine.tw. OR atomoxetine.tw. OR mirtazapine.tw. OR brofaromine.tw. OR moclobemide.tw. OR nefazodone.tw. OR venlafaxine.tw. OR citalopram.tw. OR escitalopram.tw. OR fluoxetine.tw. OR fluvoxamine.tw. OR paroxetine.tw. OR sertraline.tw.) OR dopaminergic agent*.tw. OR Dual diagnosis therapy.tw. OR Dual diagnosis treatment.tw. OR Dual Recovery Therapy.tw. OR glutaminergic agent*.tw. OR Bupropion.tw. OR citalopram.tw. OR dexamfetamine.tw. OR dexamphetamine.tw. OR duloxetine.tw. OR escitalopram.tw. OR fluoxetine.tw. OR gabapentin.tw. OR lisdexamfetamine.tw. OR Lithium.tw. OR “Long-acting” injectable.tw. OR paroxetine.tw. OR pemoline.tw. OR Pharmacological interventions.tw. OR prazosin.tw. OR pregabalin.tw. OR quetiapine.tw. OR sertraline.tw. OR Topiramate.tw. OR venlafaxine.tw. OR Vilazodone.tw. OR opioid*.tw. OR kratom.tw. OR hallucinogens.tw. OR inhalants.tw. OR toluene.tw. OR ((amyl OR butyl OR isobutyl).tw. AND nitrites.tw.) OR stimulants.tw. OR Sedatives.tw. OR Benzodiazepines.tw. OR Anthramycin.tw. OR Bromazepam.tw. OR Clonazepam.tw. OR Devazepide.tw. OR Diazepam.tw. OR Flumazenil.tw. OR Flunitrazepam.tw. OR Flurazepam.tw. OR Fentanyl.tw. OR Alprazolam.tw. OR Clonidine.tw. OR Hashish Clonidine.tw. OR Lorazepam.tw. OR Nitrazepam.tw. OR Oxazepam.tw. OR Pirenzepine.tw. OR Prazepam.tw. OR Temazepam.tw. OR Chlordiazepoxide.tw. OR Clorazepate Dipotassium.tw. OR Estazolam.tw. OR Medazepam.tw. OR Midazolam.tw. OR Triazolam.tw. OR opiate*.tw. OR Heroin.tw. OR opium.tw. OR Codeine.tw. OR Hydrocodone.tw. OR Oxycodone.tw. OR Dihydromorphine.tw. OR Ethylmorphine.tw. OR Heroin.tw. OR Hydromorphone.tw. OR Morphine.tw. OR Oxymorphone.tw. OR Thebaine.tw. OR Cocaine.tw. OR Methamphetamine*.tw. OR Benzphetamine.tw. OR anabolic steroids.tw. OR antihistamines.tw. OR nitrous oxide.tw. OR betel nut.tw. OR kava.tw. OR Ecstasy.tw. OR phenylalkylamines.tw. OR mescaline.tw. OR “2,5-dimethoxy-4-methylamphetamine”.tw. OR MDMA.tw. OR “3,4-methylenedioxymethamphetamine”.tw. OR indoleamine*.tw. OR psilocybin.tw. OR psilocin.tw. OR dimethyltryptamine.tw. OR ergoline*.tw. OR lysergic acid diethylamide.tw. OR morning glory seeds.tw. OR Salvia divinorum.tw. OR jimsonweed.tw. OR anxiolytic.tw. OR benzodiazepine*.tw. OR zolpidem.tw. OR zaleplon.tw. OR carbamate*.tw. OR glutethimide.tw. OR meprobamate.tw. OR barbiturate*.tw. OR secobarbital.tw. OR barbiturate*.tw. OR glutethimide.tw. OR methaqualone.tw. OR amphetamine.tw. OR dextroamphetamine.tw. OR methamphetamine.tw. OR gabapentin.tw. OR baclofen.tw. OR diacetylmorphine.tw. OR polydrug.tw. OR “poly-drug”.tw. OR polysubstance.tw. OR “poly-substance”.tw. OR injection drug.tw. OR (common mental disorders OR Anxiety disorders OR posttraumatic stress disorder* OR post-traumatic OR posttraumatic OR “post-traumatic” stress disorder OR PSTD OR stress related disorders OR social phobia* OR social anxiety OR SAD OR SP OR generalised anxiety disorder OR GAD OR “obsessive-compulsive*” OR obsessive compulsive disorder OR OCD OR Depression OR Depressive Disorders OR Depressive Symptoms OR Unipolar Depression OR Bipolar Depression OR Moderate Depression OR Persistent Depression OR Panic Disorder OR PD OR Panic attacks OR Specific Phobias) AND (medication* OR pharmacotherapy OR treatment).tw

**EMBASE**

'embryo transfer'/exp or 'fertilization in vitro'/exp or 'sperm injections, intracytoplasmic'/exp or 'zygote intrafallopian transfer'/exp OR 'reproductive techniques, assisted'/exp or 'insemination, artificial'/exp or 'ovulation induction'/exp OR 'insemination, artificial'/exp or 'insemination, artificial, homologous'/exp OR 'Clomiphene'/exp OR 'oocyte donation'/exp or ('in Vitro' NEAR/2 'fertili*'):ti,ab OR ('ivf' or 'icsi' or 'ZIFT'):ti,ab OR ('intracytopla*' NEAR/2 'sperm'):ti,ab OR 'zygote intrafallopian transfer*':ti,ab OR ('embryo transfer*' or 'ET'):ti,ab OR ('blastocyst' NEAR/2 'transfer*'):ti,ab OR 'assisted reproduct*':ti,ab OR 'artificial inseminat*':ti,ab OR 'iui':ti,ab OR 'intrauterine insemination*':ti,ab OR 'ovulation induc*':ti,ab OR ('ovari*' NEAR/2 'stimulat*'):ti,ab OR 'superovulat*':ti,ab OR 'ovarian hyperstimulation':ti,ab OR 'COH':ti,ab OR ('ovari*' NEAR/2 'induction'):ti,ab OR 'intra‐uterine insemination*':ti,ab OR 'clomi*':ti,ab OR 'ART':ti,ab OR (('want' or 'wanting' or 'hoping' or 'hope' or 'plan' or 'planning' or 'intend*' or 'intention' or 'contemplat*') NEAR/5 'pregnan*'):ti,ab

'Infertility'/exp OR 'male infertility'/exp OR 'Spermatozoa'/exp OR 'female infertility'/exp OR ('asthenozoospermi*' or 'oligospermi*' or 'azoospermi*'):ti,ab OR 'Asthenospermia':ti,ab OR 'Teratospermia':ti,ab OR 'subfertil*':ti,ab OR 'infertil*':ti,ab OR 'oligoasthenoteratozoospermi*':ti,ab OR 'Oligozoospermi*':ti,ab OR 'Aspermi*':ti,ab OR 'Sperm*':ti,ab OR 'semen':ti,ab

'Narcotics'/exp OR 'Analgesics, Opioid'/exp OR 'Psychotropic Drugs'/exp OR 'Central Nervous System Stimulants'/exp OR 'Amphetamines'/exp OR 'Hypnotics and Sedatives'/exp OR 'Benzodiazepines'/exp OR 'Morphine Derivatives'/exp OR 'Cocaine'/exp OR 'Methamphetamine'/exp OR 'Testosterone Congeners'/exp OR 'Psychopharmacology'/exp OR 'Antidepressive Agents'/exp OR 'Antimanic Agents'/exp OR 'Antipsychotic Agents'/exp OR (('medication*' OR 'pharmacotherapy' OR 'treatment') AND 'antidepressant*'):ti,ab OR ('selective serotonin reuptake inhibitor*':ti,ab OR 'Serotonin Uptake Inhibitors'/exp OR 'SSRIs':ti,ab OR ('serotonin' AND 'norepinephrine reuptake inhibitor*'):ti,ab OR 'SNRIs':ti,ab OR ('norepinephrine' AND 'dopamine reuptake inhibitors'):ti,ab OR 'NDRIs':ti,ab OR 'Tricyclic antidepressant*':ti,ab OR 'TCAs':ti,ab OR 'monoamine oxidase inhibitor*':ti,ab OR 'MAOIs':ti,ab) AND ('network':ti,ab OR 'mixed-treatment':ti,ab OR 'multiple treatment*':ti,ab) OR ('buspirone':ti,ab OR 'phenelzine':ti,ab OR 'atomoxetine':ti,ab OR 'mirtazapine':ti,ab OR 'brofaromine':ti,ab OR 'moclobemide':ti,ab OR 'nefazodone':ti,ab OR 'venlafaxine':ti,ab OR 'citalopram':ti,ab OR 'escitalopram':ti,ab OR 'fluoxetine':ti,ab OR 'fluvoxamine':ti,ab OR 'paroxetine':ti,ab OR 'sertraline':ti,ab) OR 'dopaminergic agent*':ti,ab OR 'Dual diagnosis therapy':ti,ab OR 'Dual diagnosis treatment':ti,ab OR 'Dual Recovery Therapy':ti,ab OR 'glutaminergic agent*':ti,ab OR 'Bupropion':ti,ab OR 'citalopram':ti,ab OR 'dexamfetamine':ti,ab OR 'dexamphetamine':ti,ab OR 'duloxetine':ti,ab OR 'escitalopram':ti,ab OR 'fluoxetine':ti,ab OR 'gabapentin':ti,ab OR 'lisdexamfetamine':ti,ab OR 'Lithium':ti,ab OR 'Long-acting injectable':ti,ab OR 'paroxetine':ti,ab OR 'pemoline':ti,ab OR 'Pharmacological interventions':ti,ab OR 'prazosin':ti,ab OR 'pregabalin':ti,ab OR 'quetiapine':ti,ab OR 'sertraline':ti,ab OR 'Topiramate':ti,ab OR 'venlafaxine':ti,ab OR 'Vilazodone':ti,ab OR 'opioid*':ti,ab OR 'kratom':ti,ab OR 'hallucinogens':ti,ab OR 'inhalants':ti,ab OR 'toluene':ti,ab OR (('amyl' OR 'butyl' OR 'isobutyl'):ti,ab AND 'nitrites':ti,ab) OR 'stimulants':ti,ab OR 'Sedatives':ti,ab OR 'Benzodiazepines':ti,ab OR 'Anthramycin':ti,ab OR 'Bromazepam':ti,ab OR 'Clonazepam':ti,ab OR 'Devazepide':ti,ab OR 'Diazepam':ti,ab OR 'Flumazenil':ti,ab OR 'Flunitrazepam':ti,ab OR 'Flurazepam':ti,ab OR 'Fentanyl':ti,ab OR 'Alprazolam':ti,ab OR 'Clonidine':ti,ab OR 'Hashish Clonidine':ti,ab OR 'Lorazepam':ti,ab OR 'Nitrazepam':ti,ab OR 'Oxazepam':ti,ab OR 'Pirenzepine':ti,ab OR 'Prazepam':ti,ab OR 'Temazepam':ti,ab OR 'Chlordiazepoxide':ti,ab OR 'Clorazepate Dipotassium':ti,ab OR 'Estazolam':ti,ab OR 'Medazepam':ti,ab OR 'Midazolam':ti,ab OR 'Triazolam':ti,ab OR 'opiate*':ti,ab OR 'Heroin':ti,ab OR 'opium':ti,ab OR 'Codeine':ti,ab OR 'Hydrocodone':ti,ab OR 'Oxycodone':ti,ab OR 'Dihydromorphine':ti,ab OR 'Ethylmorphine':ti,ab OR 'Heroin':ti,ab OR 'Hydromorphone':ti,ab OR 'Morphine':ti,ab OR 'Oxymorphone':ti,ab OR 'Thebaine':ti,ab OR 'Cocaine':ti,ab OR 'Methamphetamine*':ti,ab OR 'Benzphetamine':ti,ab OR 'anabolic steroids':ti,ab OR 'antihistamines':ti,ab OR 'nitrous oxide':ti,ab OR 'betel nut':ti,ab OR 'kava':ti,ab OR 'Ecstasy':ti,ab OR 'phenylalkylamines':ti,ab OR 'mescaline':ti,ab OR '2,5-dimethoxy-4-methylamphetamine':ti,ab OR 'MDMA':ti,ab OR '3,4-methylenedioxymethamphetamine':ti,ab OR 'indoleamine*':ti,ab OR 'psilocybin':ti,ab OR 'psilocin':ti,ab OR 'dimethyltryptamine':ti,ab OR 'ergoline*':ti,ab OR 'lysergic acid diethylamide':ti,ab OR 'morning glory seeds':ti,ab OR 'Salvia divinorum':ti,ab OR 'jimsonweed':ti,ab OR 'anxiolytic':ti,ab OR 'benzodiazepine*':ti,ab OR 'zolpidem':ti,ab OR 'zaleplon':ti,ab OR 'carbamate*':ti,ab OR 'glutethimide':ti,ab OR 'meprobamate':ti,ab OR 'barbiturate*':ti,ab OR 'secobarbital':ti,ab OR 'barbiturate*':ti,ab OR 'glutethimide':ti,ab OR 'methaqualone':ti,ab OR 'amphetamine':ti,ab OR 'dextroamphetamine':ti,ab OR 'methamphetamine':ti,ab OR 'gabapentin':ti,ab OR 'baclofen':ti,ab OR 'diacetylmorphine':ti,ab OR 'polydrug':ti,ab OR 'poly-drug':ti,ab OR 'polysubstance':ti,ab OR 'poly-substance':ti,ab OR 'injection drug':ti,ab OR ('common mental disorders' OR 'Anxiety disorders' OR 'posttraumatic stress disorder*' OR 'post-traumatic' OR 'posttraumatic' OR 'post-traumatic stress disorder' OR 'PSTD' OR 'stress related disorders' OR 'social phobia*' OR 'social anxiety' OR 'SAD' OR 'SP' OR 'generalised anxiety disorder' OR 'GAD' OR 'obsessive-compulsive*' OR 'obsessive compulsive disorder' OR 'OCD' OR 'Depression' OR 'Depressive Disorders' OR 'Depressive Symptoms' OR 'Unipolar Depression' OR 'Bipolar Depression' OR 'Moderate Depression' OR 'Persistent Depression' OR 'Panic Disorder' OR 'PD' OR 'Panic attacks' OR 'Specific Phobias') AND ('medication*' OR 'pharmacotherapy' OR 'treatment'):ti,ab

**PsycInfo**

TI ((“in Vitro” N2 “fertili*”) OR (“ivf” or “icsi” or “ZIFT”) OR (“intracytopla*” N2 “sperm”) OR “zygote intrafallopian transfer*” OR (“embryo transfer*” or “ET”) OR (“blastocyst” N2 “transfer*”) OR “assisted reproduct*” OR “artificial inseminat* OR “iui” OR “intrauterine insemination*” OR “ovulation induc*” OR (“ovari*” N2 “stimulat*”) OR “intra-uterine insemination*” OR “clomi*” OR “ART” OR ((“want” or “wanting” or “hoping” or “hope” or “plan” or “planning” or “intend*” or “intention” or “contemplat*”) N5 “pregnan*”)) OR AB ((“in Vitro” N2 “fertili*”) OR (“ivf” or “icsi” or “ZIFT”) OR (“intracytopla*” N2 “sperm”) OR “zygote intrafallopian transfer*” OR (“embryo transfer*” or “ET”) OR (“blastocyst” N2 “transfer*”) OR “assisted reproduct*” OR “artificial inseminat* OR “iui” OR “intrauterine insemination*” OR “ovulation induc*” OR (“ovari*” N2 “stimulat*”) OR “intra-uterine insemination*” OR “clomi*” OR “ART” OR ((“want” or “wanting” or “hoping” or “hope” or “plan” or “planning” or “intend*” or “intention” or “contemplat*”) N5 “pregnan*”))

AND

TI ((“asthenozoospermi*” or “oligospermi*” or “azoospermi*”) OR “Asthenospermia” OR “Teratospermia” OR “subfertil*” OR “infertil*” OR “oligoasthenoteratozoospermi*” OR “Oligozoospermi*” OR “Aspermi*” OR “Sperm*” OR “semen”) OR AB ((“asthenozoospermi*” or “oligospermi*” or “azoospermi*”) OR “Asthenospermia” OR “Teratospermia” OR “subfertil*” OR “infertil*” OR “oligoasthenoteratozoospermi*” OR “Oligozoospermi*” OR “Aspermi*” OR “Sperm*” OR “semen”)

AND

TI (((“medication*” OR “pharmacotherapy” OR “treatment”) AND “antidepressant*”) OR (“selective serotonin reuptake inhibitors” OR “Serotonin Uptake Inhibitors/” OR “SSRIs” OR (“serotonin” AND “norepinephrine reuptake inhibitor*”) OR “SNRIs” OR (“norepinephrine” AND “dopamine reuptake inhibitors”) OR “NDRIs” OR “Tricyclic antidepressant*” OR “TCAs” OR “monoamine oxidase inhibitor*” OR “MAOIs”) AND (“network” OR “mixed-treatment” OR “multiple treatment*”)) OR AB (((“medication*” OR “pharmacotherapy” OR “treatment”) AND “antidepressant*”) OR (“selective serotonin reuptake inhibitors” OR “Serotonin Uptake Inhibitors/” OR “SSRIs” OR (“serotonin” AND “norepinephrine reuptake inhibitor*”) OR “SNRIs” OR (“norepinephrine” AND “dopamine reuptake inhibitors”) OR “NDRIs” OR “Tricyclic antidepressant*” OR “TCAs” OR “monoamine oxidase inhibitor*” OR “MAOIs”) AND (“network” OR “mixed-treatment” OR “multiple treatment*”)) OR TI ((“buspirone” OR “phenelzine” OR “atomoxetine” OR “mirtazapine” OR “brofaromine” OR “moclobemide” OR “nefazodone” OR “venlafaxine” OR “citalopram” OR “escitalopram” OR “fluoxetine” OR “fluvoxamine” OR “paroxetine” OR “sertraline”)) OR AB ((“buspirone” OR “phenelzine” OR “atomoxetine” OR “mirtazapine” OR “brofaromine” OR “moclobemide” OR “nefazodone” OR “venlafaxine” OR “citalopram” OR “escitalopram” OR “fluoxetine” OR “fluvoxamine” OR “paroxetine” OR “sertraline”)) OR TI (“dopaminergic agent*” OR “Dual diagnosis therapy” OR “Dual diagnosis treatment” OR “Dual Recovery Therapy” OR “glutaminergic agent*” OR “Bupropion” OR “citalopram” OR “dexamfetamine” OR “dexamphetamine” OR “duloxetine” OR “escitalopram” OR “fluoxetine” OR “gabapentin” OR “lisdexamfetamine” OR “Lithium” OR “Long-acting injectable” OR “paroxetine” OR “pemoline” OR “Pharmacological interventions” OR “prazosin” OR “pregabalin” OR “quetiapine” OR “sertraline” OR “Topiramate” OR “venlafaxine” OR “Vilazodone”) OR AB (“dopaminergic agent*” OR “Dual diagnosis therapy” OR “Dual diagnosis treatment” OR “Dual Recovery Therapy” OR “glutaminergic agent*” OR “Bupropion” OR “citalopram” OR “dexamfetamine” OR “dexamphetamine” OR “duloxetine” OR “escitalopram” OR “fluoxetine” OR “gabapentin” OR “lisdexamfetamine” OR “Lithium” OR “Long-acting injectable” OR “paroxetine” OR “pemoline” OR “Pharmacological interventions” OR “prazosin” OR “pregabalin” OR “quetiapine” OR “sertraline” OR “Topiramate” OR “venlafaxine” OR “Vilazodone”) OR TI (“opioid*” OR “kratom” OR “hallucinogens” OR “inhalants” OR “toluene” OR ((“amyl” OR “butyl” OR “isobutyl”) AND “nitrites”) OR “stimulants” OR “Sedatives” OR “Benzodiazepines” OR “Anthramycin” OR “Bromazepam” OR “Clonazepam” OR “Devazepide” OR “Diazepam” OR “Flumazenil” OR “Flunitrazepam” OR “Flurazepam” OR “Fentanyl” OR “Alprazolam” OR “Clonidine” OR “Hashish Clonidine” OR “Lorazepam” OR “Nitrazepam” OR “Oxazepam” OR “Pirenzepine” OR “Prazepam” OR “Temazepam” OR “Chlordiazepoxide” OR “Clorazepate Dipotassium” OR “Estazolam” OR “Medazepam” OR “Midazolam” OR “Triazolam” OR “opiate*” OR “Heroin” OR “opium” OR “Codeine” OR “Hydrocodone” OR “Oxycodone” OR “Dihydromorphine” OR “Ethylmorphine” OR “Heroin” OR “Hydromorphone” OR “Morphine” OR “Oxymorphone” OR “Thebaine” OR “Cocaine” OR “Methamphetamine*” OR “Benzphetamine” OR “anabolic steroids” OR “antihistamines” OR “nitrous oxide” OR “betel nut” OR “kava” OR “Ecstasy” OR “phenylalkylamines” OR “mescaline” OR “2,5-dimethoxy-4-methylamphetamine” OR “MDMA” OR “3,4-methylenedioxymethamphetamine” OR “indoleamine*” OR “psilocybin” OR “psilocin” OR “dimethyltryptamine” OR “ergoline*” OR “lysergic acid diethylamide” OR “morning glory seeds” OR “Salvia divinorum” OR “jimsonweed” OR “anxiolytic” OR “benzodiazepine*” OR “zolpidem” OR “zaleplon” OR “carbamate*” OR “glutethimide” OR “meprobamate” OR “barbiturate*” OR “secobarbital” OR “barbiturate*” OR “glutethimide” OR “methaqualone” OR “amphetamine” OR “dextroamphetamine” OR “methamphetamine” OR “gabapentin” OR “baclofen” OR “diacetylmorphine” OR “polydrug” OR “poly-drug” OR “polysubstance” OR “poly-substance” OR “injection drug”) OR AB (“opioid*” OR “kratom” OR “hallucinogens” OR “inhalants” OR “toluene” OR ((“amyl” OR “butyl” OR “isobutyl”) AND “nitrites”) OR “stimulants” OR “Sedatives” OR “Benzodiazepines” OR “Anthramycin” OR “Bromazepam” OR “Clonazepam” OR “Devazepide” OR “Diazepam” OR “Flumazenil” OR “Flunitrazepam” OR “Flurazepam” OR “Fentanyl” OR “Alprazolam” OR “Clonidine” OR “Hashish Clonidine” OR “Lorazepam” OR “Nitrazepam” OR “Oxazepam” OR “Pirenzepine” OR “Prazepam” OR “Temazepam” OR “Chlordiazepoxide” OR “Clorazepate Dipotassium” OR “Estazolam” OR “Medazepam” OR “Midazolam” OR “Triazolam” OR “opiate*” OR “Heroin” OR “opium” OR “Codeine” OR “Hydrocodone” OR “Oxycodone” OR “Dihydromorphine” OR “Ethylmorphine” OR “Heroin” OR “Hydromorphone” OR “Morphine” OR “Oxymorphone” OR “Thebaine” OR “Cocaine” OR “Methamphetamine*” OR “Benzphetamine” OR “anabolic steroids” OR “antihistamines” OR “nitrous oxide” OR “betel nut” OR “kava” OR “Ecstasy” OR “phenylalkylamines” OR “mescaline” OR “2,5-dimethoxy-4-methylamphetamine” OR “MDMA” OR “3,4-methylenedioxymethamphetamine” OR “indoleamine*” OR “psilocybin” OR “psilocin” OR “dimethyltryptamine” OR “ergoline*” OR “lysergic acid diethylamide” OR “morning glory seeds” OR “Salvia divinorum” OR “jimsonweed” OR “anxiolytic” OR “benzodiazepine*” OR “zolpidem” OR “zaleplon” OR “carbamate*” OR “glutethimide” OR “meprobamate” OR “barbiturate*” OR “secobarbital” OR “barbiturate*” OR “glutethimide” OR “methaqualone” OR “amphetamine” OR “dextroamphetamine” OR “methamphetamine” OR “gabapentin” OR “baclofen” OR “diacetylmorphine” OR “polydrug” OR “poly-drug” OR “polysubstance” OR “poly-substance” OR “injection drug”) OR TI ((“common mental disorders” OR “Anxiety disorders” OR “posttraumatic stress disorder*” OR “post-traumatic” OR “posttraumatic” OR “post-traumatic stress disorder” OR “PSTD” OR “stress related disorders” OR “social phobia*” OR “social anxiety” OR “SAD” OR “SP” OR “generalised anxiety disorder” OR “GAD” OR “obsessive-compulsive*” OR “obsessive compulsive disorder” OR “OCD” OR “Depression” OR “Depressive Disorders” OR “Depressive Symptoms” OR “Unipolar Depression” OR “Bipolar Depression” OR “Moderate Depression” OR “Persistent Depression” OR “Panic Disorder” OR “PD” OR “Panic attacks” OR “Specific Phobias”) AND (“medication*” OR “pharmacotherapy” OR “treatment”)) OR AB ((“common mental disorders” OR “Anxiety disorders” OR “posttraumatic stress disorder*” OR “post-traumatic” OR “posttraumatic” OR “post-traumatic stress disorder” OR “PSTD” OR “stress related disorders” OR “social phobia*” OR “social anxiety” OR “SAD” OR “SP” OR “generalised anxiety disorder” OR “GAD” OR “obsessive-compulsive*” OR “obsessive compulsive disorder” OR “OCD” OR “Depression” OR “Depressive Disorders” OR “Depressive Symptoms” OR “Unipolar Depression” OR “Bipolar Depression” OR “Moderate Depression” OR “Persistent Depression” OR “Panic Disorder” OR “PD” OR “Panic attacks” OR “Specific Phobias”) AND (“medication*” OR “pharmacotherapy” OR “treatment”))

**Cochrane**

MeSH descriptor: [embryo transfer] explode all trees

MeSH descriptor: [fertilization in vitro] explode all trees

MeSH descriptor: [sperm injections, intracytoplasmic] explode all trees

MeSH descriptor: [zygote intrafallopian transfer] explode all trees

MeSH descriptor: [reproductive techniques, assisted] explode all trees

MeSH descriptor: [insemination, artificial] explode all trees

MeSH descriptor: [ovulation induction] explode all trees

MeSH descriptor: [insemination, artificial] explode all trees

MeSH descriptor: [insemination, artificial, homologous] explode all trees

MeSH descriptor: [Clomiphene] explode all trees

MeSH descriptor: [oocyte donation] explode all trees

(‘in Vitro’ NEAR/2 fertili*) OR (ivf or icsi or ZIFT) OR (intracytopla* NEAR/2 sperm) OR (zygote intrafallopian transfer*) OR (embryo transfer* or ET) OR (blastocyst NEAR/2 transfer*) OR (assisted reproduct*) OR (artificial inseminat*) OR iui OR (intrauterine insemination*) OR (ovulation induc*) OR (ovari* NEAR/2 stimulat*) OR superovulat* OR ovarian hyperstimulation OR COH OR (ovari* NEAR/2 induction) OR (intra‐uterine insemination*) OR clomi* OR ART OR ((want or wanting or hoping or hope or plan or planning or intend* or intention or contemplat*) NEAR/5 pregnan*)

MeSH descriptor: [Infertility] explode all trees

MeSH descriptor: [male infertility] explode all trees

MeSH descriptor: [Spermatozoa] explode all trees

MeSH descriptor: [female infertility] explode all trees

(asthenozoospermi* or oligospermi* or azoospermi*) OR Asthenospermia OR Teratospermia OR subfertil* OR infertil* OR oligoasthenoteratozoospermi* OR Oligozoospermi* OR Aspermi* OR Sperm* OR semen

MeSH descriptor: [Serotonin Uptake Inhibitors] explode all trees

MeSH descriptor: [Narcotics] explode all trees

MeSH descriptor: [Analgesics, Opioid] explode all trees

MeSH descriptor: [Psychotropic Drugs] explode all trees

MeSH descriptor: [Central Nervous System Stimulants] explode all trees

MeSH descriptor: [Amphetamines] explode all trees

MeSH descriptor: [Hypnotics and Sedatives] explode all trees

MeSH descriptor: [Benzodiazepines] explode all trees

MeSH descriptor: [Morphine Derivatives] explode all trees

MeSH descriptor: [Cocaine] explode all trees

MeSH descriptor: [Methamphetamine] explode all trees

MeSH descriptor: [Testosterone Congeners] explode all trees

MeSH descriptor: [Psychopharmacology] explode all trees

MeSH descriptor: [Antidepressive Agents] explode all trees

MeSH descriptor: [Antimanic Agents] explode all trees

MeSH descriptor: [Antipsychotic Agents] explode all trees

(((medication* OR pharmacotherapy OR treatment) AND antidepressant*) OR ‘selective serotonin reuptake inhibitor*’ OR SSRIs OR (serotonin AND ‘norepinephrine reuptake inhibitor*’) OR SNRIs OR (norepinephrine AND ‘dopamine reuptake inhibitors’) OR NDRIs OR ‘Tricyclic antidepressant*’ OR TCAs OR ‘monoamine oxidase inhibitor*’ OR MAOIs) AND (network OR ‘mixed treatment’ OR ‘multiple treatment*’) OR (buspirone OR phenelzine OR atomoxetine OR mirtazapine OR brofaromine OR moclobemide OR nefazodone OR venlafaxine OR citalopram OR escitalopram OR fluoxetine OR fluvoxamine OR paroxetine OR sertraline) OR (‘dopaminergic agent*’ OR ‘Dual diagnosis therapy’ OR ‘Dual diagnosis treatment’ OR ‘Dual Recovery Therapy’ OR ‘glutaminergic agent*’ OR Bupropion OR citalopram OR dexamfetamine OR dexamphetamine OR duloxetine OR escitalopram OR fluoxetine OR gabapentin OR lisdexamfetamine OR Lithium OR ‘Long-acting injectable’ OR paroxetine OR pemoline OR ‘Pharmacological interventions’ OR prazosin OR pregabalin OR quetiapine OR sertraline OR Topiramate OR venlafaxine OR Vilazodone OR opioid* OR kratom OR hallucinogens OR inhalants OR toluene OR ((amyl OR butyl OR isobutyl) AND nitrites) OR stimulants OR Sedatives OR Benzodiazepines OR Anthramycin OR Bromazepam OR Clonazepam OR Devazepide OR Diazepam OR Flumazenil OR Flunitrazepam OR Flurazepam OR Fentanyl OR Alprazolam OR Clonidine OR ‘Hashish Clonidine’ OR Lorazepam OR Nitrazepam OR Oxazepam OR Pirenzepine OR Prazepam OR Temazepam OR Chlordiazepoxide OR ‘Clorazepate Dipotassium’ OR Estazolam OR Medazepam OR Midazolam OR Triazolam OR opiate* OR Heroin OR opium OR Codeine OR Hydrocodone OR Oxycodone OR Dihydromorphine OR Ethylmorphine OR Heroin OR Hydromorphone OR Morphine OR Oxymorphone OR Thebaine OR Cocaine OR Methamphetamine* OR Benzphetamine OR anabolic steroids OR antihistamines OR ‘nitrous oxide’ OR ‘betel nut’ OR kava OR Ecstasy OR phenylalkylamines OR mescaline OR ‘2,5 dimethoxy 4 methylamphetamine’ OR MDMA OR ‘3,4 methylenedioxymethamphetamine’ OR indoleamine* OR psilocybin OR psilocin OR dimethyltryptamine OR ergoline* OR ‘lysergic acid diethylamide’ OR ‘morning glory seeds’ OR ‘Salvia divinorum’ OR jimsonweed OR anxiolytic OR benzodiazepine* OR zolpidem OR zaleplon OR carbamate* OR glutethimide OR meprobamate OR barbiturate* OR secobarbital OR barbiturate* OR glutethimide OR methaqualone OR amphetamine OR dextroamphetamine OR methamphetamine OR gabapentin OR baclofen OR diacetylmorphine OR polydrug OR ‘poly drug’ OR polysubstance OR ‘poly substance’ OR ‘injection drug’ OR ((‘common mental disorders’ OR ‘Anxiety disorders’ OR ‘posttraumatic stress disorder*’ OR ‘post traumatic’ OR ‘posttraumatic’ OR ‘post-traumatic stress disorder’ OR PSTD OR ‘stress related disorders’ OR ‘social phobia*’ OR ‘social anxiety’ OR SAD OR SP OR ‘generalised anxiety disorder’ OR GAD OR ‘obsessive-compulsive*’ OR ‘obsessive compulsive disorder’ OR OCD OR Depression OR ‘Depressive Disorders’ OR ‘Depressive Symptoms’ OR ‘Unipolar Depression’ OR ‘Bipolar Depression’ OR ‘Moderate Depression’ OR ‘Persistent Depression’ OR ‘Panic Disorder’ OR PD OR ‘Panic attacks’ OR ‘Specific Phobias’) AND (medication* OR pharmacotherapy OR treatment)))

**Scopus**

(TITLE-ABS-KEY ((“in Vitro” W/2 “fertili*”) OR (“ivf” or “icsi” or “ZIFT”) OR (“intracytopla*” W/2 “sperm”) OR “zygote intrafallopian transfer*” OR (“embryo transfer*” or “ET”) OR (“blastocyst” W/2 “transfer*”) OR “assisted reproduct*” OR “artificial inseminat*” OR “iui” OR “intrauterine insemination*” OR “ovulation induc*” OR (“ovari*” W/2 “stimulat*”) OR “intrauterine insemination*” OR “clomi*” OR “ART” OR ((“want” or “wanting” or “hoping” or “hope” or “plan” or “planning” or “intend*” or “intention” or “contemplat*”) W/5 “pregnan*”))) AND (TITLE-ABS-KEY ((“asthenozoospermi*” or “oligospermi*” or “azoospermi*”) OR “Asthenospermia” OR “Teratospermia” OR “subfertil*” OR “infertil*” OR “oligoasthenoteratozoospermi*” OR “Oligozoospermi*” OR “Aspermi*” OR “Sperm*” OR “semen”)) AND (TITLE-ABS-KEY (((“medication*” OR “pharmacotherapy” OR “treatment”) AND “antidepressant*”) OR (“selective serotonin reuptake inhibitors” OR “Serotonin Uptake Inhibitors/” OR “SSRIs” OR (“serotonin” AND “norepinephrine reuptake inhibitor*”) OR “SNRIs” OR (“norepinephrine” AND “dopamine reuptake inhibitors”) OR “NDRIs” OR “Tricyclic antidepressant*” OR “TCAs” OR “monoamine oxidase inhibitor*” OR “MAOIs”) AND (“network” OR “mixed treatment” OR “multiple treatment*”)) OR TITLE-ABS-KEY ((“buspirone” OR “phenelzine” OR “atomoxetine” OR “mirtazapine” OR “brofaromine” OR “moclobemide” OR “nefazodone” OR “venlafaxine” OR “citalopram” OR “escitalopram” OR “fluoxetine” OR “fluvoxamine” OR “paroxetine” OR “sertraline”)) OR TITLE-ABS-KEY (“dopaminergic agent*” OR “Dual diagnosis therapy” OR “Dual diagnosis treatment” OR “Dual Recovery Therapy” OR “glutaminergic agent*” OR “Bupropion” OR “citalopram” OR “dexamfetamine” OR “dexamphetamine” OR “duloxetine” OR “escitalopram” OR “fluoxetine” OR “gabapentin” OR “lisdexamfetamine” OR “Lithium” OR “Long acting injectable” OR “paroxetine” OR “pemoline” OR “Pharmacological interventions” OR “prazosin” OR “pregabalin” OR “quetiapine” OR “sertraline” OR “Topiramate” OR “venlafaxine” OR “Vilazodone”) OR TITLE-ABS-KEY (“opioid*” OR “kratom” OR “hallucinogens” OR “inhalants” OR “toluene” OR ((“amyl” OR “butyl” OR “isobutyl”) AND “nitrites”) OR “stimulants” OR “Sedatives” OR “Benzodiazepines” OR “Anthramycin” OR “Bromazepam” OR “Clonazepam” OR “Devazepide” OR “Diazepam” OR “Flumazenil” OR “Flunitrazepam” OR “Flurazepam” OR “Fentanyl” OR “Alprazolam” OR “Clonidine” OR “Hashish Clonidine” OR “Lorazepam” OR “Nitrazepam” OR “Oxazepam” OR “Pirenzepine” OR “Prazepam” OR “Temazepam” OR “Chlordiazepoxide” OR “Clorazepate Dipotassium” OR “Estazolam” OR “Medazepam” OR “Midazolam” OR “Triazolam” OR “opiate*” OR “Heroin” OR “opium” OR “Codeine” OR “Hydrocodone” OR “Oxycodone” OR “Dihydromorphine” OR “Ethylmorphine” OR “Heroin” OR “Hydromorphone” OR “Morphine” OR “Oxymorphone” OR “Thebaine” OR “Cocaine” OR “Methamphetamine*” OR “Benzphetamine” OR “anabolic steroids” OR “antihistamines” OR “nitrous oxide” OR “betel nut” OR “kava” OR “Ecstasy” OR “phenylalkylamines” OR “mescaline” OR “2,5 dimethoxy 4 methylamphetamine” OR “MDMA” OR “3,4 methylenedioxymethamphetamine” OR “indoleamine*” OR “psilocybin” OR “psilocin” OR “dimethyltryptamine” OR “ergoline*” OR “lysergic acid diethylamide” OR “morning glory seeds” OR “Salvia divinorum” OR “jimsonweed” OR “anxiolytic” OR “benzodiazepine*” OR “zolpidem” OR “zaleplon” OR “carbamate*” OR “glutethimide” OR “meprobamate” OR “barbiturate*” OR “secobarbital” OR “barbiturate*” OR “glutethimide” OR “methaqualone” OR “amphetamine” OR “dextroamphetamine” OR “methamphetamine” OR “gabapentin” OR “baclofen” OR “diacetylmorphine” OR “polydrug” OR “poly drug” OR “polysubstance” OR “poly substance” OR “injection drug”) OR TITLE-ABS-KEY ((“common mental disorders” OR “Anxiety disorders” OR “posttraumatic stress disorder*” OR “post-traumatic” OR “posttraumatic” OR “post traumatic stress disorder” OR “PSTD” OR “stress related disorders” OR “social phobia*” OR “social anxiety” OR “SAD” OR “SP” OR “generalised anxiety disorder” OR “GAD” OR “obsessive compulsive*” OR “obsessive compulsive disorder” OR “OCD” OR “Depression” OR “Depressive Disorders” OR “Depressive Symptoms” OR “Unipolar Depression” OR “Bipolar Depression” OR “Moderate Depression” OR “Persistent Depression” OR “Panic Disorder” OR “PD” OR “Panic attacks” OR “Specific Phobias”) AND (“medication*” OR “pharmacotherapy” OR “treatment”)))

**Web of Science**

TI=((“in Vitro” NEAR/2 “fertili*”) OR (“ivf” or “icsi” or “ZIFT”) OR (“intracytopla*” NEAR/2 “sperm”) OR “zygote intrafallopian transfer*” OR (“embryo transfer*” or “ET”) OR (“blastocyst” NEAR/2 “transfer*”) OR “assisted reproduct*” OR “artificial inseminat* OR “iui” OR “intrauterine insemination*” OR “ovulation induc*” OR (“ovari*” NEAR/2 “stimulat*”) OR “intra-uterine insemination*” OR “clomi*” OR “ART” OR ((“want” or “wanting” or “hoping” or “hope” or “plan” or “planning” or “intend*” or “intention” or “contemplat*”) NEAR/5 “pregnan*”)) OR AB=((“in Vitro” NEAR/2 “fertili*”) OR (“ivf” or “icsi” or “ZIFT”) OR (“intracytopla*” NEAR/2 “sperm”) OR “zygote intrafallopian transfer*” OR (“embryo transfer*” or “ET”) OR (“blastocyst” NEAR/2 “transfer*”) OR “assisted reproduct*” OR “artificial inseminat* OR “iui” OR “intrauterine insemination*” OR “ovulation induc*” OR (“ovari*” NEAR/2 “stimulat*”) OR “intra-uterine insemination*” OR “clomi*” OR “ART” OR ((“want” or “wanting” or “hoping” or “hope” or “plan” or “planning” or “intend*” or “intention” or “contemplat*”) NEAR/5 “pregnan*”))

AND

TI=((“asthenozoospermi*” or “oligospermi*” or “azoospermi*”) OR “Asthenospermia” OR “Teratospermia” OR “subfertil*” OR “infertil*” OR “oligoasthenoteratozoospermi*” OR “Oligozoospermi*” OR “Aspermi*” OR “Sperm*” OR “semen”) OR AB=((“asthenozoospermi*” or “oligospermi*” or “azoospermi*”) OR “Asthenospermia” OR “Teratospermia” OR “subfertil*” OR “infertil*” OR “oligoasthenoteratozoospermi*” OR “Oligozoospermi*” OR “Aspermi*” OR “Sperm*” OR “semen”)

AND

TI=(((“medication*” OR “pharmacotherapy” OR “treatment”) AND “antidepressant*”) OR (“selective serotonin reuptake inhibitors” OR “Serotonin Uptake Inhibitors/” OR “SSRIs” OR (“serotonin” AND “norepinephrine reuptake inhibitor*”) OR “SNRIs” OR (“norepinephrine” AND “dopamine reuptake inhibitors”) OR “NDRIs” OR “Tricyclic antidepressant*” OR “TCAs” OR “monoamine oxidase inhibitor*” OR “MAOIs”) AND (“network” OR “mixed-treatment” OR “multiple treatment*”)) OR AB=(((“medication*” OR “pharmacotherapy” OR “treatment”) AND “antidepressant*”) OR (“selective serotonin reuptake inhibitors” OR “Serotonin Uptake Inhibitors/” OR “SSRIs” OR (“serotonin” AND “norepinephrine reuptake inhibitor*”) OR “SNRIs” OR (“norepinephrine” AND “dopamine reuptake inhibitors”) OR “NDRIs” OR “Tricyclic antidepressant*” OR “TCAs” OR “monoamine oxidase inhibitor*” OR “MAOIs”) AND (“network” OR “mixed-treatment” OR “multiple treatment*”)) OR TI=((“buspirone” OR “phenelzine” OR “atomoxetine” OR “mirtazapine” OR “brofaromine” OR “moclobemide” OR “nefazodone” OR “venlafaxine” OR “citalopram” OR “escitalopram” OR “fluoxetine” OR “fluvoxamine” OR “paroxetine” OR “sertraline”)) OR AB=((“buspirone” OR “phenelzine” OR “atomoxetine” OR “mirtazapine” OR “brofaromine” OR “moclobemide” OR “nefazodone” OR “venlafaxine” OR “citalopram” OR “escitalopram” OR “fluoxetine” OR “fluvoxamine” OR “paroxetine” OR “sertraline”)) OR TI=(“dopaminergic agent*” OR “Dual diagnosis therapy” OR “Dual diagnosis treatment” OR “Dual Recovery Therapy” OR “glutaminergic agent*” OR “Bupropion” OR “citalopram” OR “dexamfetamine” OR “dexamphetamine” OR “duloxetine” OR “escitalopram” OR “fluoxetine” OR “gabapentin” OR “lisdexamfetamine” OR “Lithium” OR “Long-acting injectable” OR “paroxetine” OR “pemoline” OR “Pharmacological interventions” OR “prazosin” OR “pregabalin” OR “quetiapine” OR “sertraline” OR “Topiramate” OR “venlafaxine” OR “Vilazodone”) OR AB=(“dopaminergic agent*” OR “Dual diagnosis therapy” OR “Dual diagnosis treatment” OR “Dual Recovery Therapy” OR “glutaminergic agent*” OR “Bupropion” OR “citalopram” OR “dexamfetamine” OR “dexamphetamine” OR “duloxetine” OR “escitalopram” OR “fluoxetine” OR “gabapentin” OR “lisdexamfetamine” OR “Lithium” OR “Long-acting injectable” OR “paroxetine” OR “pemoline” OR “Pharmacological interventions” OR “prazosin” OR “pregabalin” OR “quetiapine” OR “sertraline” OR “Topiramate” OR “venlafaxine” OR “Vilazodone”) OR TI=(“opioid*” OR “kratom” OR “hallucinogens” OR “inhalants” OR “toluene” OR ((“amyl” OR “butyl” OR “isobutyl”) AND “nitrites”) OR “stimulants” OR “Sedatives” OR “Benzodiazepines” OR “Anthramycin” OR “Bromazepam” OR “Clonazepam” OR “Devazepide” OR “Diazepam” OR “Flumazenil” OR “Flunitrazepam” OR “Flurazepam” OR “Fentanyl” OR “Alprazolam” OR “Clonidine” OR “Hashish Clonidine” OR “Lorazepam” OR “Nitrazepam” OR “Oxazepam” OR “Pirenzepine” OR “Prazepam” OR “Temazepam” OR “Chlordiazepoxide” OR “Clorazepate Dipotassium” OR “Estazolam” OR “Medazepam” OR “Midazolam” OR “Triazolam” OR “opiate*” OR “Heroin” OR “opium” OR “Codeine” OR “Hydrocodone” OR “Oxycodone” OR “Dihydromorphine” OR “Ethylmorphine” OR “Heroin” OR “Hydromorphone” OR “Morphine” OR “Oxymorphone” OR “Thebaine” OR “Cocaine” OR “Methamphetamine*” OR “Benzphetamine” OR “anabolic steroids” OR “antihistamines” OR “nitrous oxide” OR “betel nut” OR “kava” OR “Ecstasy” OR “phenylalkylamines” OR “mescaline” OR “2,5-dimethoxy-4-methylamphetamine” OR “MDMA” OR “3,4-methylenedioxymethamphetamine” OR “indoleamine*” OR “psilocybin” OR “psilocin” OR “dimethyltryptamine” OR “ergoline*” OR “lysergic acid diethylamide” OR “morning glory seeds” OR “Salvia divinorum” OR “jimsonweed” OR “anxiolytic” OR “benzodiazepine*” OR “zolpidem” OR “zaleplon” OR “carbamate*” OR “glutethimide” OR “meprobamate” OR “barbiturate*” OR “secobarbital” OR “barbiturate*” OR “glutethimide” OR “methaqualone” OR “amphetamine” OR “dextroamphetamine” OR “methamphetamine” OR “gabapentin” OR “baclofen” OR “diacetylmorphine” OR “polydrug” OR “poly-drug” OR “polysubstance” OR “poly-substance” OR “injection drug”) OR AB=(“opioid*” OR “kratom” OR “hallucinogens” OR “inhalants” OR “toluene” OR ((“amyl” OR “butyl” OR “isobutyl”) AND “nitrites”) OR “stimulants” OR “Sedatives” OR “Benzodiazepines” OR “Anthramycin” OR “Bromazepam” OR “Clonazepam” OR “Devazepide” OR “Diazepam” OR “Flumazenil” OR “Flunitrazepam” OR “Flurazepam” OR “Fentanyl” OR “Alprazolam” OR “Clonidine” OR “Hashish Clonidine” OR “Lorazepam” OR “Nitrazepam” OR “Oxazepam” OR “Pirenzepine” OR “Prazepam” OR “Temazepam” OR “Chlordiazepoxide” OR “Clorazepate Dipotassium” OR “Estazolam” OR “Medazepam” OR “Midazolam” OR “Triazolam” OR “opiate*” OR “Heroin” OR “opium” OR “Codeine” OR “Hydrocodone” OR “Oxycodone” OR “Dihydromorphine” OR “Ethylmorphine” OR “Heroin” OR “Hydromorphone” OR “Morphine” OR “Oxymorphone” OR “Thebaine” OR “Cocaine” OR “Methamphetamine*” OR “Benzphetamine” OR “anabolic steroids” OR “antihistamines” OR “nitrous oxide” OR “betel nut” OR “kava” OR “Ecstasy” OR “phenylalkylamines” OR “mescaline” OR “2,5-dimethoxy-4-methylamphetamine” OR “MDMA” OR “3,4-methylenedioxymethamphetamine” OR “indoleamine*” OR “psilocybin” OR “psilocin” OR “dimethyltryptamine” OR “ergoline*” OR “lysergic acid diethylamide” OR “morning glory seeds” OR “Salvia divinorum” OR “jimsonweed” OR “anxiolytic” OR “benzodiazepine*” OR “zolpidem” OR “zaleplon” OR “carbamate*” OR “glutethimide” OR “meprobamate” OR “barbiturate*” OR “secobarbital” OR “barbiturate*” OR “glutethimide” OR “methaqualone” OR “amphetamine” OR “dextroamphetamine” OR “methamphetamine” OR “gabapentin” OR “baclofen” OR “diacetylmorphine” OR “polydrug” OR “poly-drug” OR “polysubstance” OR “poly-substance” OR “injection drug”) OR TI=((“common mental disorders” OR “Anxiety disorders” OR “posttraumatic stress disorder*” OR “post-traumatic” OR “posttraumatic” OR “post-traumatic stress disorder” OR “PSTD” OR “stress related disorders” OR “social phobia*” OR “social anxiety” OR “SAD” OR “SP” OR “generalised anxiety disorder” OR “GAD” OR “obsessive-compulsive*” OR “obsessive compulsive disorder” OR “OCD” OR “Depression” OR “Depressive Disorders” OR “Depressive Symptoms” OR “Unipolar Depression” OR “Bipolar Depression” OR “Moderate Depression” OR “Persistent Depression” OR “Panic Disorder” OR “PD” OR “Panic attacks” OR “Specific Phobias”) AND (“medication*” OR “pharmacotherapy” OR “treatment”)) OR AB=((“common mental disorders” OR “Anxiety disorders” OR “posttraumatic stress disorder*” OR “post-traumatic” OR “posttraumatic” OR “post-traumatic stress disorder” OR “PSTD” OR “stress related disorders” OR “social phobia*” OR “social anxiety” OR “SAD” OR “SP” OR “generalised anxiety disorder” OR “GAD” OR “obsessive-compulsive*” OR “obsessive compulsive disorder” OR “OCD” OR “Depression” OR “Depressive Disorders” OR “Depressive Symptoms” OR “Unipolar Depression” OR “Bipolar Depression” OR “Moderate Depression” OR “Persistent Depression” OR “Panic Disorder” OR “PD” OR “Panic attacks” OR “Specific Phobias”) AND (“medication*” OR “pharmacotherapy” OR “treatment”))

Supplementary Material, List S3. List of papers awaiting classification.

1. Worly, B. L., & Gur, T. L. (2015). The effect of mental illness and psychotropic medication on gametes and fertility: a systematic review. The Journal of clinical psychiatry, 76(7), 5625.
2. Volgsten, H. (2013). Prevalence of depressive disorders in infertile women and men up to 5 years after undergoing in vitro fertilization treatment na follow-up study. In Human Reproduction (Vol. 28, No. S1, pp. 280-281).
3. Rapakko, S., & Menke, M. (2015). Factors associated with baseline stress levels at time of referral to an infertility clinic [160]. Obstetrics & Gynecology, 125, 55S.
4. Moreno, A. & Roca, M. & Quiron, G.H.. (2009). Pregnancy and assisted reproduction technologies. Revista Iberoamericana de Fertilidad y Reproduccion Humana. 26. 25-33.
5. Katayama, K. P., Roesler, M., Gunnarson, C., Stehlik, E., Jagusch, S., & Meyer, M. A. (1988). Ultrasound-guided transvaginal needle aspiration of follicles for in vitro fertilization. Obstetrics & Gynecology, 72(2), 271-274.
6. Gehad, A., Weidenmuller, M., & Sharma, J. (2021, June). A case report of an extremely rare complication of ovarian hyperstimulation. In BJOG-AN INTERNATIONAL JOURNAL OF OBSTETRICS AND GYNAECOLOGY (Vol. 128, pp. 239-240). 111 RIVER ST, HOBOKEN 07030-5774, NJ USA: WILEY.
7. Freeman, M. P., Toth, T. L., & Cohen, L. S. (2013). Assisted reproduction and risk of depressive relapse: considerations for treatment. , 25, 4, 25(4), 283-288.
8. Christensen, P., Cloherty, J., Ryan, H., Downer, R., Conway, U., Lowe, E., ... & Parner, E. (2017, July). Intervention to reduce sperm DNA damage prior to fertility treatment. In Human Reproduction (Vol. 32, pp. 170-171). GREAT CLARENDON ST, OXFORD OX2 6DP, ENGLAND: OXFORD UNIV PRESS.
9. Brownridge, S. R., & Rausch, M. (2021). ARE FERTILITY TREATMENT RETENTION RATES AFFECTED BY PATIENT PSYCHIATRIC HISTORY?. Fertility and Sterility, 116(3), e71.
10. Akyuz, A., Gurhan, N., & Atici, D. (2006). The effect of male and female depression-anxiety levels on oocyte-sperm number and pregnancy rates during IVF treatment.
11. Episodes of anxiety affect ICSI outcomes. (2005). Reproductive biomedicine online, 10(3), 389.
12. Figueras-Puigderrajols, N., Ballesteros, A., & Guerra, D. (2021). P–478 Quality of life assessment in women undergoing assisted reproduction. A study of FertiQol and HADS. Human Reproduction, 36(Supplement_1), deab130-477.
13. Schneider, H.P.G. (2000). 198. Tagung der Niederrheinisch-Westfalischen Gesellschaft fur Gynakologie und Geburtshilfe. 28. bis 29. Mai 1999, Munster. Zentralblatt fur Gynakologie, 122 (6), 334-349.

Supplementary Material, List S4. List of excluded studies with reason.

Wrong context (8):

1. Evans-Hoeker, E. A., Eisenberg, E., Legro, R. S., Diamond, M. P., & Steiner, A. Z. (2017). Depressive symptoms, antidepressant use and fertility treatment outcomes. Fertility and Sterility, 108(3), e298-e299.
2. Wildt, L., Sir-Petermann, T., Leyendecker, G., Waibel-Treber, S., & Rabenbauer, B. (1993). Opiate antagonist treatment of ovarian failure. Human Reproduction, 8(suppl_2), 168-174.
3. Markoula, S., Siarava, E., Kostoulas, C., Zikopoulos, A., & Georgiou, I. (2020). An open study of valproate in subfertile men with epilepsy. Acta Neurologica Scandinavica, 142(4), 317-322.
4. Knight, J. C., Pandit, A. S., Rich, A. M., Trevisani, G. T., & Rabinowitz, T. (2015). Clomiphene-associated suicide behavior in a man treated for hypogonadism: case report and review of the literature. Psychosomatics, 56(5), 598-602.
5. Evans-Hoeker, E. A., Eisenberg, E., Diamond, M. P., Legro, R. S., Alvero, R., Coutifaris, C., ... & Witter, F. (2018). Major depression, antidepressant use, and male and female fertility. Fertility and sterility, 109(5), 879-887.
6. Shahine, L. K., & Lathi, R. B. (2006). Night sweats and elevated follicle-stimulating hormone levels while taking selective serotonin reuptake inhibitors. Obstetrics & Gynecology, 108(3 Part 2), 741-742.
7. Persaud, R. N., & Lam, R. W. (1998). Manic reaction after induction of ovulation with gonadotropins. American Journal of Psychiatry, 155(3), 447-a.
8. Yonkers, K. A. (2021). Treatment of psychiatric conditions in pregnancy starts with planning. American Journal of Psychiatry, 178(3), 213-214.

Wrong concept (3):

1. Youssef, M. A., Van Wely, M., Hassan, M. A., Al-Inany, H. G., Mochtar, M., Khattab, S., & Van der Veen, F. (2010). Can dopamine agonists reduce the incidence and severity of OHSS in IVF/ICSI treatment cycles? A systematic review and meta-analysis. Human Reproduction Update, 16(5), 459-466.
2. Masoumi, S. Z., Parsa, P., Kalhori, F., Mohagheghi, H., & Mohammadi, Y. (2019). What psychiatric interventions are used for anxiety disorders in infertile couples? A systematic review study. Iranian Journal of Psychiatry, 14(2), 160.
3. Shapira SC, Chrubasik S, Hoffmann A, et al. Use of alfentanil for in vitro fertilization oocyte retrieval. J Clin Anesth. 1996;8:282–285.

Supplementary Material, List S5. List of studies which we labeled as ongoing studies

1. Schmidt, L., Hageman, I., Hougaard, C. Ø., Sejbaek, C. S., Assens, M., Ebdrup, N. H., & Pinborg, A. (2013). Psychiatric disorders among women and men in assisted reproductive technology (ART) treatment. The Danish National ART-Couple (DANAC) cohort: protocol for a longitudinal, national register-based cohort study. BMJ open, 3(3), e002519.
2. Comparison the influence of Fluoxetine with midwifery counseling in depressed women treated for infertility. <https://trialsearch.who.int/Trial2.aspx?TrialID=IRCT2014031116911N2>
3. The Effect of Selective Serotonin Reuptake Inhibitor (SSRI) Treatment on Affective Symptoms and Fertility Treatment Outcome in Women Undergoing in Vitro Fertilization (IVF). <https://clinicaltrials.gov/show/NCT00989053>
4. AMIR, H., et al. P–441 Semen quality and cryopreservation in adolescent transgender females. Human Reproduction, 2021, 36.Supplement_1: deab130. 440.
5. RHODES, Q.; BOWMAN, R.; NODLER, J. PILOT INVESTIGATION ON THE IMPACT OF AMPHETAMINE STIMULANT EXPOSURE ON OOCYTE MATURITY, FERTILIZATION AND BLASTOCYST DEVELOPMENT OUTCOMES. Fertility and Sterility, 2023, 120.1: e65-e66.
6. KASSI, Luce A., et al. PSYCHOLOGICAL DISTRESS IN WOMEN CONSIDERING OR UNDERGOING FERTILITY TREATMENTS DURING THE COVID-19 PANDEMIC. Fertility and Sterility, 2021, 116.3: e361.
7. KASSI, Luce A., et al. PSYCHOLOGICAL DISTRESS IN WOMEN CONSIDERING OR UNDERGOING FERTILITY TREATMENTS DURING THE OMICRON SURGE OF THE COVID-19 PANDEMIC. Fertility and Sterility, 2022, 118.4: e313.
8. HERNANDEZ-NIETO, C. A., et al. Selective serotonin reuptake inhibitors exposure prior to art treatment does not affect blastulation rate. Fertility and Sterility, 2017, 108.3: e93.
9. DOMAR, A. D., et al. The prevalence of antidepressant use by women undergoing IVF. Fertility and Sterility, 2012, 98.3: S45.
10. DOMAR, Alice D., et al. THE PSYCHOLOGICAL IMPACT OF THE COVID-19 PANDEMIC ON WOMEN PREGNANT FOLLOWING ART: A LONGITUDINAL STUDY. Fertility and Sterility, 2021, 116.3: e364.
11. YANG, C., et al. A comparison of anxiety and depression in IVF patients and oocyte donors. Fertility and Sterility, 2018, 110.4: e67.
12. KNIGHT, Anna K., et al. Association of mental health diagnoses and uterine endometrial thickness in women undergoing in-vitro fertilization. Fertility and Sterility, 2019, 112.3: e377.

Supplementary Material, Table S6. Map of evidence from the identified reviews.

| Review ID | Primary studies | | | | | | | | | | | | | | | | | | | | | | | | | | |
| --- | --- | --- | --- | --- | --- | --- | --- | --- | --- | --- | --- | --- | --- | --- | --- | --- | --- | --- | --- | --- | --- | --- | --- | --- | --- | --- | --- |
|  | 1 | 2 | 3 | 4 | 5 | 6 | 7 | 8 | 9 | 10 | 11 | 12 | 13 | 14 | 15 | 16 | 17 | 18 | 19 | 20 | 21 | 22 | 23 | 24 | 25 | 26 | 27 |
| Milosavljević 2022 | 1 | 1 | 1 | 1 |  |  |  |  |  |  |  |  |  |  |  |  |  |  |  |  |  |  |  |  |  |  |  |
| Williams 2007 |  | 1 |  |  |  |  |  |  |  |  |  |  |  |  |  |  |  |  |  |  |  |  |  |  |  |  |  |
| Wilkins 2010 |  |  |  |  | 1 | 1 | 1 |  |  |  |  |  |  |  |  |  |  |  |  |  |  |  |  |  |  |  |  |
| Sylvester 2019 | 1 | 1 | 1 | 1 |  |  |  |  |  | 1 | 1 | 1 |  |  |  |  |  |  |  |  |  |  |  |  |  |  |  |
| Stanhiser 2018 |  |  |  |  |  |  |  |  |  |  | 1 |  | 1 |  |  |  |  |  |  |  |  |  |  |  |  |  |  |
| Riddle 2023 |  |  |  |  |  |  |  | 1 |  |  |  |  |  | 1 |  |  |  |  |  |  |  |  |  |  |  |  |  |
| Holka-Pokorska 2015 |  | 1 |  |  |  |  |  |  |  |  |  |  |  |  |  |  |  |  |  |  |  |  |  |  |  |  |  |
| Becker 2019 | 1 |  | 1 | 1 |  |  |  |  |  |  |  |  |  |  | 1 |  | 1 |  |  |  |  |  |  |  |  |  |  |
| Domar 2017 |  |  |  |  |  |  |  |  | 1 |  |  |  |  |  |  | 1 |  | 1 |  |  |  |  |  |  |  |  |  |
| Akioyamen 2016 | 1 | 1 |  | 1 |  |  |  |  |  | 1 |  |  |  |  |  |  |  |  |  |  |  |  |  |  |  |  |  |
| Roussos-Ross 2018 |  |  |  |  |  |  |  |  |  | 1 |  |  |  |  |  |  | 1 | 1 |  |  |  |  |  |  |  |  |  |
| Vlahos 2009 |  |  |  |  |  |  |  |  |  |  |  |  |  |  |  |  |  |  | 1 | 1 |  |  | 1 |  |  |  |  |
| Van de Velde 2005 |  |  |  |  |  |  |  |  |  |  |  |  |  |  |  |  |  |  |  |  | 1 | 1 | 1 |  |  |  |  |
| Tsen 2007 |  |  |  |  |  |  |  |  |  |  |  |  |  |  |  |  |  |  |  |  | 1 | 1 | 1 | 1 | 1 | 1 | 1 |
| Domar 2013 | 1 | 1 |  | 1 |  |  | 1 | 1 |  | 1 |  |  |  |  |  |  |  |  |  |  |  |  |  |  |  |  |  |
| Rymaszewska 2019 |  |  |  |  |  |  |  |  |  |  |  |  |  |  |  |  |  |  |  |  |  |  |  |  |  |  |  |
| 1=Serafini 2009; 2=Klock 2004;3=Hernandez-Nieto 2017;4=Friedman 2009;5=Warnock 1998; 6=Noorbala 2008; 7=Faramrazi 2008a; 8=Faramarzi 2008b; 9=Faramrazi 2013; 10=Ramezanzadehet 2011; 11=Cesta 2016; 12=Evans-Hoeker 2018; 13=Evans-Hoeker 2017; 14=Aisenberg 2019; 15=Wang 2006; 16=Domar 2013; 17=Nillni 2016; 18=Domar 2015; 19=Ditkoff 1997; 20=Sterzik 1994; 21=Wilhelm 2002; 22=Casati 1999; 23=Ben-Shlomo 1999; 24=Bruce 1985; 25=Shapira 1996; 26=Cardasis 1976; 27=Swanson 1992. | | | | | | | | | | | | | | | | | | | | | | | | | | | |

List of included reviews:

1. Milosavljević, J. Z., Milosavljević, M. N., Arsenijević, P. S., Milentijević, M. N., & Stefanović, S. M. (2022). The effects of selective serotonin reuptake inhibitors on male and female fertility: a brief literature review. International journal of psychiatry in clinical practice, 26(1), 43-49.
2. Williams, K. E., Marsh, W. K., & Rasgon, N. L. (2007). Mood disorders and fertility in women: a critical review of the literature and implications for future research. Human Reproduction Update, 13(6), 607-616.
3. Wilkins, K. M., Warnock, J. K., & Serrano, E. (2010). Depressive symptoms related to infertility and infertility treatments. Psychiatric Clinics, 33(2), 309-321.
4. Sylvester, C., Menke, M., & Gopalan, P. (2019). Selective serotonin reuptake inhibitors and fertility: considerations for couples trying to conceive. Harvard Review of Psychiatry, 27(2), 108-118.
5. Stanhiser, J., & Steiner, A. Z. (2018). Psychosocial aspects of fertility and assisted reproductive technology. Obstetrics and Gynecology Clinics, 45(3), 563-574.
6. Riddle, J. N., Hopkins, T., Yeaton-Massey, A., & Hellberg, S. (2023). No baby to bring home: perinatal loss, infertility, and mental illness—overview and recommendations for care. Current Psychiatry Reports, 25(11), 747-757.
7. Holka-Pokorska, J., Jarema, M., & Wichniak, A. (2015). Clinical determinants of mental disorders occurring during the infertility treatment. Psychiatr Pol, 49(5), 965-982.
8. Becker, M. A., Chandy, A., Mayer, J. L., Sachdeva, J., Albertini, E. S., Sham, C., & Worley, L. L. (2019). Psychiatric aspects of infertility. American Journal of Psychiatry, 176(9), 765-766.
9. Domar, A. D. (2017). The mind/body connection. In The Boston IVF Handbook of Infertility (pp. 202-210). CRC Press.
10. Akioyamen, L. E., Minhas, H., Holloway, A. C., Taylor, V. H., Akioyamen, N. O., & Sherifali, D. (2016). Effects of depression pharmacotherapy in fertility treatment on conception, birth, and neonatal health: a systematic review. Journal of psychosomatic research, 84, 69-80.
11. Roussos-Ross, D., Rhoton-Vlasak, A. S., Baker, K. M., Arkerson, B. J., & Graham, G. (2018). Case-based care for pre-existing or new-onset mood disorders in patients undergoing infertility therapy. Journal of Assisted Reproduction and Genetics, 35, 1371-1376.
12. Vlahos, N. F., Giannakikou, I., Vlachos, A., & Vitoratos, N. (2009). Analgesia and anesthesia for assisted reproductive technologies. International Journal of Gynecology & Obstetrics, 105(3), 201-205.
13. Van de Velde, M. (2005). Anesthesia for in-vitro fertilization. Current Opinion in Anesthesiology, 18(4), 428-430.
14. Tsen, L. C. (2007). Anesthesia for assisted reproductive technologies. International Anesthesiology Clinics, 45(1), 99-113.
15. Domar AD, Moragianni VA, Ryley DA, Urato AC. The risks of selective serotonin reuptake inhibitor use in infertile women: A review of the impact on fertility, pregnancy, neonatal health and beyond. Hum Reprod 2013;28:160 –71
16. Rymaszewska, J., Szczesniak, D., Cubała, W. J., Gałecki, P., Rybakowski, J., Samochowiec, J., & Dudek, D. (2019). Recommendations of the Polish Psychiatric Association for treatment of affective disorders in women of childbearing age. Part III: Approach to pregnancy loss and unsuccessful in vitro treatment of infertility. Psychiatr. Pol, 53(2), 277-292.

List of identified primary studies from reviews :

1. Serafini P, Lobo DS, Grosman A, et al: Fluoxetine treatment for anxiety in women undergoing in vitro fertilization. Int J Gynaecol Obstet 2009; 105:136–139 Crossref. PubMed.
2. Klock S, Sheinin S, Kazer R, Zhang X. A pilot study of the relationship between selective sero- tonin reuptake inhibitors and in vitro fertilization outcome. Fertil. Steril. 2004; 82 (4): 968–969.
3. Hernandez-Nieto, C., Lee, J., Nazem, T., Gounko, D., Copperman, A., & Sandler, B. (2017). Embryo aneuploidy is not impacted by selective serotonin reuptake inhibitor exposure. Fertility and Sterility, 108(6), 973-979.
4. Friedman, B. E., Rogers, J. L., Shahine, L. K., Westphal, L. M., & Lathi, R. B. (2009). Effect of selective serotonin reuptake inhibitors on in vitro fertilization outcome. Fertility and sterility, 92(4), 1312-1314.
5. Warnock JK, Bundren JC, Morris DW. Depressive mood symptoms associated with ovarian suppression. Fertil Steril 2000;74(5):984–6.
6. Noorbala AA, Ramazanzadeh F, Malekafzali H, et al. Effects of a psychological intervention on depression in infertile couples. Int J Gynaecol Obstet 2008;101:248–52.
7. Faramarzi M, Alipor A, Esmaelzadeh S, et al. Treatment of depression and anxiety in infertile women: cognitive behavioral therapy versus fluoxetine. J Affect Disord 2008;108(1–2):159–64.
8. Faramarzi M, Kheirkhah F, Esmaelzadeh S, Alipour A, Hjiahmadi M, Rahnama J. Is psychotherapy a reliable alternative to pharmacother- apy to promote the mental health of infertile women? A randomized clinical trial. Eur J Obstet Gynecol Reprod Biol. 2008;141(1):49–53.
9. Faramarzi M, Pasha H, Esmailzadah S, Kheirkhah F, Heidary S, Afshar Z. The effect of the cognitive behavior therapy and pharmacotherapy on infertility stress: A randomized controlled trial. Int J Fertil Steril 2013;7:199–206
10. Ramezanzadeh, F., Noorbala, A. A., Abedinia, N., Forooshani, A. R., & Naghizadeh, M. M. (2011). Psychiatric intervention improved pregnancy rates in infertile couples. The Malaysian journal of medical sciences: MJMS, 18(1), 16.
11. Cesta, C. E., Viktorin, A., Olsson, H., Johansson, V., Sjölander, A., Bergh, C., ... & Iliadou, A. N. (2016). Depression, anxiety, and antidepressant treatment in women: association with in vitro fertilization outcome. Fertility and sterility, 105(6), 1594-1602.
12. Evans-Hoeker, E. A., Eisenberg, E., Diamond, M. P., Legro, R. S., Alvero, R., Coutifaris, C., ... & Witter, F. (2018). Major depression, antidepressant use, and male and female fertility. Fertility and sterility, 109(5), 879-887.
13. Evans-Hoeker, E. A., Eisenberg, E., Legro, R. S., Diamond, M. P., & Steiner, A. Z. (2017). Depressive symptoms, antidepressant use and fertility treatment outcomes. Fertility and Sterility, 108(3), e298-e299.
14. Aisenberg Romano G, Fried Zaig I, Halevy A, Azem F, Amit A, Bloch M. Prophylactic SSRI treatment for women suffering from mood and anxiety symptoms undergoing in vitro fertilization—a prospective placebo-controlled study. Arch Womens Ment Health. 2019;22(4):503–10.
15. Wang H, Dey SK, Maccarrone M: Jekyll and Hyde: two faces of cannabinoid signaling in male and female fertility. Endocr Rev 2006; 27:427–448 Crossref. PubMed.
16. Domar AD, Moragianni VA, Ryley DA, Urato AC. The risks of selective serotonin reuptake inhibitor use in infertile women: A review of the impact on fertility, pregnancy, neonatal health and beyond. Hum Reprod 2013;28:160 –71
17. Nillni YI, Wesselink AK, Gradus JL, Hatch EE, Rothman KJ, Mikkelsen EM, et al. Depression, anxiety, and psychotropic medi- cation use and fecundability. Am J Obstet Gynecol. 2016;215(4): 453.e1–8.
18. Domar AD, Gross J, Rooney K, Boivin J. Exploratory randomized trial on the effect of a brief psychological intervention on emotions, quality of life, discontinuation, and pregnancy rates in in vitro fer- tilization patients. Fertil Steril. 2015;104(2):440–51. e7
19. Ditkoff EC, Plumb J, Selick A, Sauer MV. Anesthesia practices in the United States common to in vitro fertilization (IVF) centers. J Assist Reprod Genet 1997;14(3):145–7.
20. Sterzik K, Nitsch CD, Korda P, Sasse V, Rosenbusch B, Marx T, et al. The effect of different anesthetic procedures on hormone levels in women. Studies during an in vitro fertilization-embryo transfer (IVF-ET) program [in German]. Anaesthesist 1994;43(11):738–42.
21. Wilhelm W, Hammadeh ME, White PF, et al. General anesthesia versus monitored anesthesia care with remifentanil for assisted reproductive tech- nologies: effect on pregnancy rate. J Clin Anesth 2002; 14:1–5.
22. Casati A, Valentini G, Zangrillo A, et al. Anaesthesia for ultrasound guided oocyte retrieval: midazolam/remifentanil versus propofol/fentanyl regimens. Eur J Anaesthesiol 1999; 16:773–778.
23. Ben-Shlomo I, Moskovich R, Katz Y, Shalev E. Midazolam/ketamine sedative combination compared with fentanyl/propofol/isoflurane anaesthesia for oocyte retrieval. Hum Reprod 1999; 14:1757–1759.
24. Bruce DL, Hinkley R, Norman PF. Fentanyl does not inhibit fertilization or early development of sea urchin eggs. Anesth Analg. 1985;64:498–500.
25. Shapira SC, Chrubasik S, Hoffmann A, et al. Use of alfentanil for in vitro fertilization oocyte retrieval. J Clin Anesth. 1996;8:282–285.
26. Cardasis C, Schuel H. The sea urchin egg as a model system to study the effects of narcotics on secretion. In: Ford DH, Clouet DH, eds. Tissue Responses to Addictive Drugs. New York: Spectrum; 1976:631–640.
27. Swanson RF, Leavitt MG. Fertilization and mouse embryo development in the presence of midazolam. Anesth Analg. 1992;75:549–554.

Supplementary Material, Table S7. General characteristics of included studies (N=29).

| Name of the first author | Year of publication | Country of corresponding authors | Study design | Funding | COI | Ethical approval | written/any consent obtained from participants | Type of ART | Reason for ART | Number of people | Age | % of female |
| --- | --- | --- | --- | --- | --- | --- | --- | --- | --- | --- | --- | --- |
| Rodrigues, J. D. M. | 2014 | Portugal | CR | NR | No | NR | Yes | IVF | uterine pathology (secondary infertility) | 1 | 40 | 100 |
| Ng, E. H. Y. | 2002 | China | RCT | NR | No | obtained | Yes | ICSI | primary infertility, tuboperitoneal factor, male factor, endometriosis, unexplained or mixed causes | 100 | median 35,5 2.5–97.5th centiles 26-42.2 | 100 |
| McIntosh, M. D. | 2010 | USA | CR | NR | No | NR | NR | IVF | unsuccesful trials to conceive | 1 | 48 | 100 |
| Keshavarz, S. | 2020 | Iran | RCT | NR | No | obtained | Yes | IVF | female/male factor, both, idiopathic | 60 | fluoxetine group - mean 30.47 SD 4.57; control group - mean 30.43 SD 5.34 | 100 |
| Goisis, A. | 2023 | UK | Cohort | Non-industry | NS | obtained | NR | ICSI;IVF,FET | NR | 575921 | range 20-45 | 100 |
| Friedman, B. E. | 2009 | USA | CC | NR | NS | obtained | NR | ICSI;IVF | NR | 950 | mean SSRI users 40.1 non-SSRI users 38.7 | 100 |
| Applegarth, L. D. | 2015 | USA | CR | NR | No | NR | NR | IVF | the limitations of fertility, given the age | 1 | 42 | 100 |
| Walker, Z. | 2023 | USA | CS | None | NS | obtained | NR | ICSI | Diminihsed ovarian reserve, uterine factor, ovulatory dysfunction, tubal factor, endometrosis, male factor, unexplained, other | 222 | mean 37.38 SD 4.90 | 0 |
| Hviid Malling, G. M. | 2020 | Denmark | Obs | None | NS | only considered | NR | ICSI;IVF, FET,egg donation | NR | 138464 | NR | 100 |
| Siegel A | 2018 | USA | CR | NR | NS | only considered | NR | IVF | infertility | 1 | 37 | 100 |
| Hernandez-Nieto C | 2017 | USA | Cohort | NR | NS | obtained | NR | IVF | infertility | 4335 | not exposed: mean 37 SD 4.4; exposed 37 SD 4.7 | 100 |
| Carvalho C | 2021 | Brazil | CS | Non-industry | No | obtained | Yes | NR | endometriosis;tubal factor;male factor;PCOS | 90 | mean 35.89 SD 5.20 | 100 |
| Ramezanzadeh F | 2010 | Iran | RCT | Non-industry | No | NR | Yes | IVF | male;female;both;unexplained | 280 | women: treatment mean 26.57 SD 4.14, control mean 26.09 SD 4.69; men: treatment mean 30.99 SD 4.42, control mean 31.24 SD 5.56 | 50 |
| Keshavarz S | 2020 | Iran | RCT | Non-industry | NS | obtained | Yes | IVF | NR | 74 | control mean 32.29 SD 5.73, fluoxetine mean 30.19 SD 5.02, midwifery consultation mean 30.0 SD 4.17 | 100 |
| Sylvester C. | 2020 | USA | CS | NR | No | obtained | Yes | IVF | NR | 72 | mean 34.4 SD 3.8 | 100 |
| Merkan S. | 2005 | Turkey | CR | NR | No | NR | NR | ICSI | male infertility of 10 years’ duration | 1 | 30 | 100 |
| Lu S. | 2009 | China | CaS | Non-industry | NS | NR | NR | ICSI,IVF | fallopian obstruction | 2 | mean 32,5 | 0 |
| Pedro J. | 2019 | Portugal | Cohort | Non-industry | NS | obtained | NR | NR | NR | 1009 | mean 31.78 SD 3.63 | 100 |
| Elkington N. | 2003 | UK | CS | NR | No | NR | NR | IVF | NR | NA | NA | NA |
| Roest I. | 2019 | the Netherlands | CS | None | No | NR | NR | ICSI,IVF | NR | NA | NA | NA |
| Volgsten, H. | 2008 | Sweden | CS | Non-industry | No | obtained | Yes | ICSI;IVF | female factor;male factor;unexplained;other | 1090 | females: mean 32.9 SD 3.9; males: mean 34.7 SD 4.8 | 50 |
| Serafini, P. | 2009 | Brazil | RCT | NR | No | obtained | Yes | IVF | combined male and female factors;endometriosis;tubal disease;ovulatory causes;male factor;idiopathic infertility | 152 | fluoxetine group (FLX) - mean 33.5 SD 3.1; follicle acid group (FA) - mean 33.8 SD 3.6 | 100 |
| Klock, S. C. | 2004 | USA | CS | NR | No | obtained | NR | IVF | ovulatory dysfunction;unexplained;tubal factor;endometriosis;PCOS | 75 | mean 36 | 100 |
| Casati, A. | 1999 | Italy | RCT | Non-industry | No | obtained | Yes | IVF | NR | 60 | the midazolam/remifentanil group - mean 35 SD 3 years; the propofol/fentanyl group - mean 34 SD 4 years | 100 |
| Ben-Shlomo, I. | 1999 | Israel | RCT | NR | No | obtained | Yes | ICSI | NR | 50 | anaesthesia group - mean 34.2 SD 5.6; sedation group - mean 34.2 SD 6.5 | 100 |
| Aisenberg Romano, G. | 2019 | Israel | RCT | Non-industry | NS | obtained | Yes | IVF | female factor;male factor;both;unexplained | 41 | mean 44.60 SD 3.25 (treatment group); mean 41.83 SD 4.28 (placebo group); dispersion 18-45 | 100 |
| Karibe J. | 2024 | Japan | CR | None | No | only considered | Yes | ICSI | ejaculatory disorder | 1 | 32 | 0 |
| CS=Cross-sectional; RCT=randomised controlled trials; CC=case-control;Obs=observational;CR=case report; CaS=case series; NR=not reported; NS=non significant;  * transgender females before initiating hormone therapy | | | | | | | | | | | | |

Supplementary Material, Table S8. Types of outcomes in included studies (N=29).

| Name of the first author | Year of publication | Outcomes related to ART | | | | Outcomes related to birth | | | Outcomes related to mental health | |
| --- | --- | --- | --- | --- | --- | --- | --- | --- | --- | --- |
|  |  | Semen | Cycle | Efficacy | other | live birth rate | multiple pregnancy rate | other | severity of symptoms | other |
| Rodrigues, J. D. M. | 2014 | - | - | - | - | - | - | - | psychotic^1^ | - |
| Ng, E. H. Y. | 2002 | - | Number of oocytes | retrieval rate | No. of follicles punctured;TUGOR duration (min) | - | - | - | anxiety level^2^ | Sedation level^3^;satisfaction level;postoperative side effects |
| McIntosh, M. D. | 2010 | - | - | - | - | - | - | - | depression^4^ | - |
| Keshavarz, S. | 2020 | - | Number of oocytes | Fertilization rate (%) | number of harvested oocytes | - | - | - | depression^5^ | TNF-α levels;cortisol levels |
| Goisis, A. | 2023 | - | - | - | - | - | - | - | - | - |
| Friedman, B. E. | 2009 | - | Number of oocytes | Fertilization rate (%);Number of usable blastocysts;number of oocytes retrieved;cycle cancellation rate | - | Yes | - | spontaneous abortion rate | - | - |
| Applegarth, L. D. | 2015 | - | - | - | - | - | - | - | - | - |
| Walker, Z. | 2023 | Initial total volume (ml), Initial concentration (M/ml), Initial motility (%), Initial total motile sperm count, Final total volume (ml), Final concentration M/ml, Final motility (%), Final total motile sperm count | - | - | - | - | - | - | - | - |
| Hviid | 2020 | - | - | - | number of initiated ART | Yes | - | - | - | - |
| Siegel A | 2018 | - | - | - | - | - | - | - | - | - |
| Hernandez-Nieto C | 2017 | - | - | Number of euploid embryos, implantation rate;clinical pregnancy rate (defined as the ratio of the number of gestational sacs (determined by ultrasound at 9 days after a positive pregnancy test) to the number of transferred embryos | - | - | Yes | early pregnancy loss | - | - |
| Carvalho C | 2021 | - | - | - | - | - | - | - | - | - |
| Ramezanzadeh F | 2010 | - | - | pregnancy | - | - | - |  | depression^5^ | - |
| Keshavarz S | 2020 | - | Number of oocytes | pregnancy | - | - | - | abortion | depression score^5^; state anxiety, trait anxiety^6^ | cortisol level |
| Sylvester C. | 2020 | - | - | positive conception | - | Yes | - | - | eating^7^, depression^8^, anxiety^9^ | - |
| Merkan S. | 2005 | - | - | - | - | - | - | - | delirium^1^ | - |
| Lu S. | 2009 | Initial total motile sperm count, Final total motile sperm count, semen retrieval | - | Fertilization rate (%) | - | - | - | - | - | - |
| Pedro J. | 2019 | - | - | - | - | - | - | - | - | relation between stress, antidepressants and delivering birth |
| Elkington N. | 2003 | - | - | - | - | - | - | - | - | - |
| Roest I. | 2019 | - | - | - | - | - | - | - | - | - |
| Volgsten, H. | 2008 | - | - | - | - | - | - | - | major depressive disorder, dysthymia, partial remission of major depress- ive disorder, GAD, panic disorder, obsessive-compulsive disorder, social phobia and bulimia nervosa^10^ | - |
| Serafini, P. | 2009 | - | - | Fertilization rate (%);Number of embryos biopsied;oocytes with two pronuclei,top quality embryos present,implantation rate | - | Yes | - | spontaneous abortion rate | anxiety^6^ | - |
| Klock, S. C. | 2004 | - | Number of oocytes | Fertilization rate (%); Blastulation rate (%);Number of embryos biopsied,peak E2 levels; percentage of zygotes developing to 8-cell embryos,pregnancy; | day of transfer;day 13 hCG value;day 15 hCG value | - | - | miscarriage rate | - | - |
| Casati, A. | 1999 | - | - | - | result of oocyte retrieval procedure | - | - | - | - | incidence of side effects; patient satisfaction; surgeon satisfaction;time taken to achieve Aldrete score=10,mental status at the post-anaesthesia care unit admittance |
| Ben-Shlomo, I. | 1999 | - | Number of oocytes | Fertilization rate (%), pregnancies; retrieved oocytes | - | - | - | - | - | oxygen saturation; response to pain; postoperative side effects;patient's overall satisfaction |
| Aisenberg Romano, G. | 2019 | - | - | - | - | - | - | - | anxiety,depression^11^ | - |
| Karibe J. | 2024 | Final total motile sperm count | - | Fertilization rate (%) | - | - | - | - | - | - |
| 1 assessed by a psychiatrist  2 measured with Chinese version of State Anxiety Questionnaire, 100mm linear visual analogue scale (VAS)  3 measured with Ramsay’s scale (Ramsay, M.A.E., Savege, T.M., Simpson, B.R.J. and Goodwin, R. (1974) Controlled sedation with alphaxalone-alphadolone. Br. Med. J., 2, 656–659.)  4 unknown way to measure  5 measured with Beck Depression Inventory (BDI)  6 measured with State-Trait Anxiety Inventory (STAI)  7 measured with The Eating Disorder Examination Questionnaire (EDE-Q),  8 measured with The Patient Health Questionnaire (PHQ-4)  9 measured with two questions from the General Anxiety Disorder assessment tool (GAD-7)  10 measured with The Primary Care Evaluation of Mental Disorders (PRIME-MD), based on the Diagnostic and Statistical Manual of Mental Disorders  11 measured with The Zung Self-Rating Anxiety Scale, Center of Epidemiologic Studies Depression Scale (CES-D), Mental Health Inventory | | | | | | | | | | |
